# Participation of the Olfactory Bulb in Circadian Organization during Early Postnatal Life in Rabbits

**DOI:** 10.1371/journal.pone.0156539

**Published:** 2016-06-15

**Authors:** Erika Navarrete, Juan Roberto Ortega-Bernal, Lucero Trejo-Muñoz, Georgina Díaz, Rodrigo Montúfar-Chaveznava, Ivette Caldelas

**Affiliations:** 1 Instituto de Investigaciones Biomédicas, Universidad Nacional Autónoma de México, Ciudad de México, México; 2 Facultad de Ingeniería, Universidad Nacional Autónoma de México, Ciudad de México, México; Université Lyon, FRANCE

## Abstract

Experimental evidence indicates that during pre-visual stages of development in mammals, circadian regulation is still not under the control of the light-entrainable hypothalamic pacemaker, raising the possibility that the circadian rhythmicity that occurs during postnatal development is under the control of peripheral oscillators, such as the main olfactory bulb (MOB). We evaluated the outcome of olfactory bulbectomy on the temporal pattern of core body temperature and gross locomotor activity in newborn rabbits. From postnatal day 1 (P1), pups were randomly assigned to one of the following conditions: intact pups (INT), intact pups fed by enteral gavage (INT+ENT), sham operated pups (SHAM), pups with unilateral lesions of the olfactory bulb (OBx-UNI), and pups with bilateral lesions of the olfactory bulb (OBx-BI). At the beginning of the experiment, from P1-8, the animals in all groups were fed at 11:00, from P9-13 the feeding schedule was delayed 6 h (17:00), and finally, from P14-15 the animals were subjected to fasting conditions. The rabbit pups of the INT, INT+ENT, SHAM and OBx-UNI groups exhibited a clear circadian rhythmicity in body temperature and locomotor activity, with a conspicuous anticipatory rise hours prior to the nursing or feeding schedule, which persisted even during fasting conditions. In addition, phase delays in the nursing or feeding schedule induced a clear phase shift in both parameters. In contrast, the OBx-BI group exhibited atypical rhythmicity in both parameters under entrained conditions that altered the anticipatory component, as well as deficient phase control of both rhythms. The present results demonstrate that the expression of circadian rhythmicity at behavioral and physiological levels during early stages of rabbit development largely depends on the integrity of the main olfactory bulb.

## Introduction

In addition to the processing of volatile chemical stimuli, the olfactory system coordinates multiple integrative functions, such as physiological regulation, emotional responses, and reproductive and social behaviors (review in [1]). Recent studies have also implicated this system in the regulation of circadian rhythmicity in mammals [2–4].

In mammals, circadian (24-h) fluctuations are evident in numerous biological processes, ranging from the behavioral to the molecular level. This temporal organization is mediated by a central circadian pacemaker located in the suprachiasmatic nucleus (SCN) and by peripheral oscillators that are present in different tissues such as liver, spleen, kidney, heart, lung and other brain structures, such as the olfactory bulb, retina, amygdala and other hypothalamic areas [2–7]. To ensure a coherent rhythmicity at the organismal level, peripheral oscillators are synchronized by the central pacemaker by a complex and redundant combination of neurochemical and hormonal signals (review in [8]). It is well known that the functional properties of SCN neurons, as well as peripheral oscillators, rely on the transcriptional–translational negative feedback loop of a set of core clock components known as “clock genes”, which drive the cyclical fluctuations in RNA transcription and protein synthesis associated with clock genes. The core clock genes include the brain and muscle aryl hydrocarbon receptor nuclear translocator-like 1 (*Bmal1*), circadian locomotor output cycles kaput (*Clock*), period (*Per 1–3*) and cryptochrome (*Cry 1–2*) genes, among others [7, 9–13].

Experimental findings indicate that the main olfactory bulb (MOB) functions as an independent circadian pacemaker in adult rodents, in a similar manner to the SCN, because MOB cells exhibit autonomous rhythmicity in their molecular profile and firing rate *in vitro* [2–4], time-regulated responses to odorants [14], and their removal produces alterations in the period of free-running rhythms of temperature and locomotor activity, delays the onset of activity and the re-entrainment rates to light-dark cycles, and modifies the diurnal rhythm of corticosterone and gonadotropin [15–19].

During early postnatal stages of development, rodents and lagomorphs exhibit clear circadian fluctuations in different variables, and it is possible to entrain their rhythmicity to external cyclical cues, indicating that the circadian system of altricial mammals is already functional [20, 21]. One remarkable example is the European rabbit (*Oryctolagus cuniculus*), since newborn rabbits exhibit circadian rhythmicity at behavioral [22, 23], physiological [23–25]], metabolic [21, 26] and molecular levels [27–29]. This is due in large part to the unusual limited pattern of maternal care, in nature, rabbits give birth in a dark burrow, in which the doe constructs a nest of dried grass and fur from her own body. Immediately after giving birth, the doe leaves the nest and only returns briefly to nurse the pups for 3–4 minutes, once every 24 hours [30, 31]. Thus, during early development rabbit pups live in complete darkness and largely isolated from potentially entraining environmental signals other than the vital, once-daily nursing visit of their mother, that acts as a potent non-photic entraining cue of the newborn pups’ circadian system. Recent studies demonstrate that it is possible to entrain pups rhythmicity using maternal volatile olfactory cues such as mammary pheromone 2-methylbut-2-enal [21], which is capable of modulating the temporal expression of clock proteins in the central pacemaker the SCN of rabbit pups [32]. This model provides an unusually good opportunity to study the early development of circadian function and non-photic synchronization, without disturbing the normal mother-young relationship or the development of the newborn (reviews [33–35]).

With regard to the generation of circadian rhythms, the molecular clockwork in the SCN is not fully mature during the first days of life in rabbits [27, 28], suggesting that circadian temporal regulation during pre-visual stages of development is still not under the control of the hypothalamic pacemaker. Moreover, bilateral SCN lesions do not disrupt circadian rhythmicity in newborn rabbits because the pups continue to display the circadian-regulated anticipatory rise to nursing in locomotor activity and core body temperature, which persists endogenously even when the nursing event is omitted [36]. This observation suggests that circadian rhythmicity during postnatal development is under the control of peripheral oscillators such as the MOB.

To address this issue, we assessed the influence of the MOB on temporal regulation in rabbit pups, and we found that complete removal of the olfactory bulb caused important alterations in the expression of circadian rhythmicity and its modulation by maternal cues, both at physiological and behavioral levels.

## Material and Methods

The experiments were performed according to the National Institutes of Health Guide for the Care and Use of Laboratory Animals (NIH Pub. No. 86–23, revised 1996) and the guidelines for the Treatment of Animals in Research of the Instituto de Investigaciones Biomédicas, Universidad Nacional Autónoma de México (UNAM). The protocol was reviewed and approved by the Animal Care and Use Committee of the Instituto de Investigaciones Biomédicas, UNAM, prior to conducting the study (Permit Number: 139). Relevant details were followed to ameliorate animal suffering.

### Animals

The chinchilla strain of domestic rabbits (*Oryctolagus cuniculus*) was used in this study. The animals were bred and maintained at the animal care facilities of the Instituto de Investigaciones Biomédicas, UNAM, as previously described [21]. Briefly, pregnant rabbits were housed in individual stainless steel cages and maintained on a 16-h light/8-h dark cycle (lights turned on at 08:00). The room temperature was maintained at 20 ± 2°C, with a relative humidity between 40 and 60%. Rabbit chow (Conejos Ganador, Malta Cleyton, México) and water were provided *ad libitum*. Four days before the programmed date of parturition, an artificial burrow made of opaque polyvinyl chloride was placed in the cage containing the pregnant rabbit. Sterile hay was placed in each maternal cage for nest building.

The day of parturition was defined as postnatal day 0 (P0). The newborn rabbits were weighed at birth, color-marked on their ears for individual identification and allowed to remain in the burrow with the mother for 6 h. Forty rabbit pups obtained from 15 litters were used. The pups were allocated to one of the following groups (n = 8): intact pups (INT), intact pups fed by enteral gavage (INT+ENT), sham operated pups (SHAM), pups with unilateral lesions of the olfactory bulb (OBx-UNI), and pups with bilateral lesions of the olfactory bulb (OBx-BI).

To avoid the presence of maternal signals at different time during the cycle, newborn rabbits were transferred to a recording room that was isolated from the rest of the colony and placed in a box in groups of four pups. The boxes were made of translucent polysulfide (23 x 13 x 15 cm), and paper towel strips and corncob bedding (Argo, México) were placed inside the box as nest material. Throughout the experiment, rabbit pups were maintained under constant light conditions (170 lux measured at the top of the box using a YK-10LX light meter, Lutron, Electronic Enterprise Co., Taiwan) at room temperature with a relative humidity as described above.

At the beginning of the experiment, from P1-8, the animals in all groups were fed at 11:00, from P9-13 the feeding schedule was delayed 6 h (17:00), and finally from P14-15 the animals were subjected to fasting conditions.

### Feeding procedure

Before performing the surgical procedures, from P0-3, the animals in all groups were fed by maternal nursing. Considering that pups in the OBx-BI group were unable to orient themselves to the lactating female and suck after total removal of the olfactory bulbs, it was necessary to feed the animals artificially. Therefore, beginning at P4, two feeding strategies were used, namely, normal maternal nursing and orogastric gavage.

The body weights of all animals in the study were obtained before and after the feeding procedure. To perform this procedure, prior to feeding, the urogenital area of the pups was gently rubbed with a moist cotton swab to produce defecation and urination, and then the body weight of the pups was immediately measured.

The procedure used for maternal nursing with a lactating doe in the INT, SHAM and OBx-UNI groups, was performed as described in previous reports [27–29]. Briefly, newborn rabbits were transferred every 24 h to the colony room and placed inside the nest box for five min. Immediately after nursing, the pups were removed from the nest, weighed and placed in their boxes.

The pups in the OBx-BI group and an additional control group (INT+ENT) were fed using the same procedure (orogastric gavage). Briefly, from P4-13, OBx-BI pups were placed inside the nest box with a lactating female for five min with their littermates, and the inability to suck, as well as a lack of milk in the stomach and no changes in body weight, were used as indicators of total removal of both olfactory bulbs. The INT+ENT pups were placed inside the nest box with a non-lactating female with nipples impregnated with the maternal pheromone 2-metyl-but-2-enal (2MB-2; 20 μl, ≥96% Aldrich, USA) for five min to emulate the odorant and other maternal cues that are present during nursing. Immediately after this period, the pups in both groups were gently immobilized, maintaining the head and body in a vertical position for oral gavage, and milk formula was slowly perfused into the stomach of the pups using a Silastic tube (0.058 in I.D. and 0.077 in O.D., Dow Corning, USA) coupled to a 20-ml syringe and a 16-G needle [21]. Only highly trained personnel performed the enteral nutrition. The time spent to feed each pup was approximately 2–4 min. Immediately after feeding, the pups were weighed and returned to their boxes in the recording room. The percentage of milk ingested was similar to previous reports [37, 38].

### Surgical procedures

#### Transponder implantation

To record individual rabbit locomotor activity and body temperature rhythms at P3 before performing the olfactory bulbectomy procedure, pups (50–70 g) were anesthetized with 5% isoflurane (SufloranVet, Pisa Agropecuaria, México) in oxygen and then maintained with 2–3% isoflurane throughout the procedure (Table Top Laboratory Animal Anesthesia System, Vetequip, INC, USA). Under aseptic conditions, the pups were implanted i.p. with a transponder (G2 E-Mitter, VitalView System, MiniMitter Respironics, Inc., USA). The duration of this procedure was generally less than five minutes.

#### Olfactory bulbectomy

The procedure was performed as described by different investigators in rabbits and rodents [39–41]. Immediately after transponder implantation, using the same anesthetic procedure, the animals were placed in a ventral decubitus on a heating pad, and the skull was exposed by generating a midline incision above the olfactory bulbs. A 5-mm trepan was drilled (Cordless Rotatory Tool, Dremel, USA) through the cribriform plate, approximately 1.5 mm anterior to the rostral dorsal cerebral vein and 2 mm lateral to the midline of the skull to expose the underlying olfactory bulb tissue, using a sterile no. 9909 tungsten carbide cutter (Dremel, USA). For the sham olfactory bulbectomy surgery, the incision was closed at this point using a sterile hemostatic sponge (Gelfoam, Pfizer, USA), and the scalp was sutured. For the unilateral and bilateral bulbectomies, one or both bulbs, respectively, were aspirated with a sterile P-1000 pipetman tip (Molecular BioProducts, USA) connected to a vacuum line, after complete removal of the tissue. The incision was then closed, and the scalp was sutured. After surgery, the animals were injected with a single dose of flunixin meglumine (2.2 mg/kg, Napzin®, Pisa Agropecuaria, México) for postsurgical analgesia, and Enrofloxacin (5 mg/kg, Enroxil 5%®, Senosiain, México) to avoid postsurgical infection.

The animals were allowed to recover from both surgeries for two days before the beginning of the physiological and behavioral recordings. For this procedure, the pups were transferred to the recording room and placed in their boxes, and telemetry was performed (ER-4000 Energizer Receiver, MiniMitter Respironics, Inc., USA). The data for both parameters were collected in 2-min bins from P6 to P15 using a VitalView telemetry system (Respironics, MiniMitter, Inc., USA) according to a previously described procedure [23].

At the end of the experiment on P16, the rabbits were deeply anesthetized and killed. The brains were removed and frozen in isopentane, and 50-μm sagittal sections were prepared through the OB and stained using the Nissl method to histologically assess the unilateral and bilateral bulbectomies.

### Data analysis

Time series obtained for both parameters, core body temperature and gross locomotor activity, were divided into three segments that were analyzed separately. The first segment consisted of P6-8 when the animals were fed at 11:00 am, the second segment comprised P9-13 when the feeding schedule was delayed 6 h (17:00), and the third segment was from P14-15 when the animals were maintained under fasting conditions.

To evaluate the phase, period, duration and intensity of the anticipatory component in the overt rhythmicity of rabbits, we used a procedure previously reported by Trejo-Muñoz et al. [23].

To evaluate the anticipatory component, it was defined as a sustained increase in the temperature or activity variables over time that crossed over or occurred during a previously defined threshold. Due to the effect of the developmental increase in temperature (or decrease in activity), we defined the threshold value as the mean of a 24-h data segment. To quantify the anticipatory component, we employed the data corresponding to the first 5 h of each 24-h segment to determine the points at which the increment began and ended (duration) using the maximum and minimum values as well as the difference between both extreme values (intensity).

Finally, the means and standard errors were calculated for daily body weight, locomotor activity, core body temperature, phases, and the duration and intensity of the anticipatory component. The differences associated with a group or postnatal day were evaluated using two-way ANOVA (for further information see S1 and S2 Texts) for repeated measures followed by the Scheffe post-hoc test (significance level of 5%).

## Results

The histological examination confirmed the group assignment (S1 Fig). In some cases, minor damage to the frontal cortex was observed. At the behavioral level, the animals in the INT, SHAM, OBx-UNI and INT+ENT groups exhibited a pattern of head searching and oral grasping nipple searching behaviors that are typical of newborn rabbits in the presence of a lactating doe or a non-lactating female impregnated with the mammary pheromone 2MB-2 [42, 43]. In contrast, the pups in the OBx-BI group did not exhibited a stereotypical pattern of nipple searching behavior with complete elimination of suckling.

### Body weight

The 2-way ANOVA revealed a significant effect of age among the groups on the body weight of newborn rabbits (Group: F_4, 468_ = 30.5; *p* = < 0.0001; Age: F_13, 468_ = 114.9; *p* = < 0.0001; Interaction: F_52, 468_ = 2.6; *p* = < 0.0001). At P1, the body weights of the animals in the INT, INT+ENT, SHAM, OBx-UNI and OBx-BI groups were similar (71.9 ± 15.8 g, 80 ± 13.4 g, 66.2 ± 8.5 g, 69.4 ± 12.1g, 67.5 ± 18.3 g, respectively). The body weight of the pups in the INT, SHAM and OBx-UNI groups increased gradually, exhibiting similar weights on P13 (185.1 ± 44.3 g, 162.4 ± 10.3 g and 175.7 ± 24.4 g, respectively). In contrast, from P10, the pups that were fed artificially in the INT+ENT and OBx-BI groups exhibited a significant difference in weight, reaching a final weight on P13 of 130 ± 20.7 g and 117.1 ± 23.1 g, respectively (S2 Fig). A significant lack of body weight gain of approximately 24–33% was observed in the newborn rabbits that received enteral nutrition compared with the INT, SHAM and OBx-UNI groups with normal nursing.

### Core body temperature

**Fig 1:**
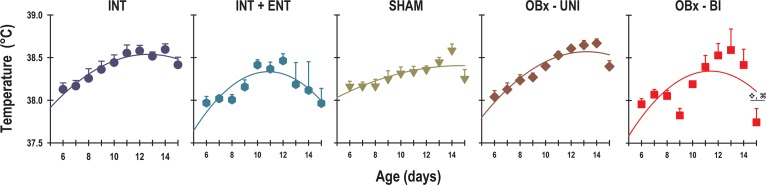
Daily average core body temperatures. The mean temperature from postnatal day 6–15 in intact rabbit pups (INT, dark blue circles), intact pups fed by enteral gavage (INT+ENT, dark cyan hexagons), sham operated pups (SHAM, dark green triangles), pups with lesions of the olfactory bulb (OBx-UNI, dark red diamonds), and pups with bilateral lesions of the olfactory bulb (OBx-BI, red squares). Mean ± SEM, r^2^ = 0.95. ❖⌘ indicates a significant difference (p<0.05) *vs*. INT and OBx-UNI.

A 2-way ANOVA revealed significant effects associated with experimental manipulation and age on the average daily temperature of newborn rabbits (Group: F_4, 348_ = 11.1; *p* = < 0.0001; Age: F_9, 348_ = 13.9; *p* = < 0.0001; Interaction: F_36, 348_ = 1.3; *p* = NS). The average daily core body temperature of the rabbit pups displayed a significant increase with age (Fig 1). The mean temperature of the INT pups on P6 was 38.1 ± 0.07°C, which increased gradually to reach 38.4 ± 0.09°C on P15. Similarly, the newborn rabbits in the INT+ENT, SHAM and OBx-UNI groups exhibited an initial core temperature on P6 of 37.9 ± 0.07°C, 38.2 ± 0.06°C and 38 ± 0.07°C, respectively. At the end of the experiment on P15, the temperature was 37.9 ± 0.18°C, 38.3 ± 0.10°C and 38.4 ± 0.06°C, respectively. In contrast, on P6, the pups in the OBx-BI group showed an initial core temperature of 37.9 ± 0.06°C and reached a final temperature of 37.7 ± 0.16°C on P15. Therefore, OBx-BI pups had a lower core body temperature, by approximately 0.7°C at the end of the experiment, compared with the pups in the INT and OBx-UNI groups (Fig 1).

**Fig 2:**
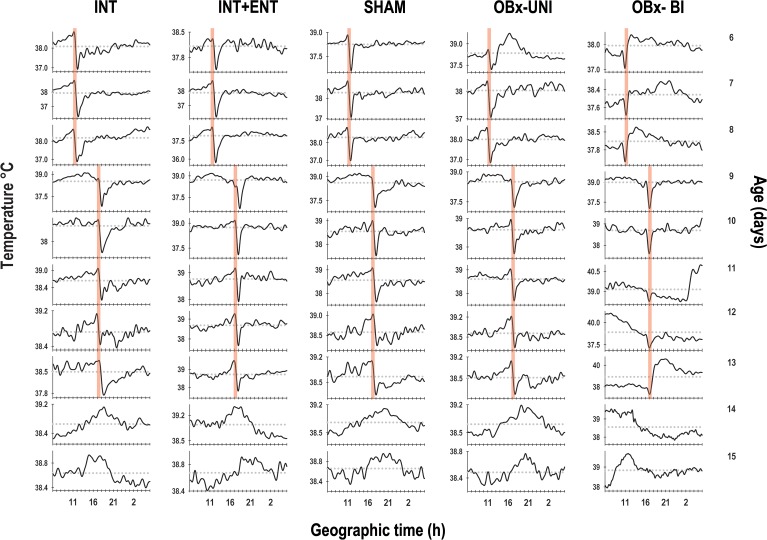
Temporal patterns of core body temperature. Representative daily profiles of temperature as measured using biotelemetry in newborn intact rabbit pups (INT), intact pups fed by enteral gavage (INT+ENT), sham operated pups (SHAM), pups with unilateral lesions of the olfactory bulb (OBx-UNI) and pups with bilateral lesions of the olfactory bulb (OBx-BI). From postnatal day 6–8, the pups were nursed or fed artificially every 24 h at 11:00 (indicated by the vertical red bar), from postnatal day 9–13 the feeding schedule was delayed 6 h (17:00), and from P14-15 the pups were maintained under fasting conditions (see text for details). The dotted red line indicates the daily mean temperature.

**Fig 3:**
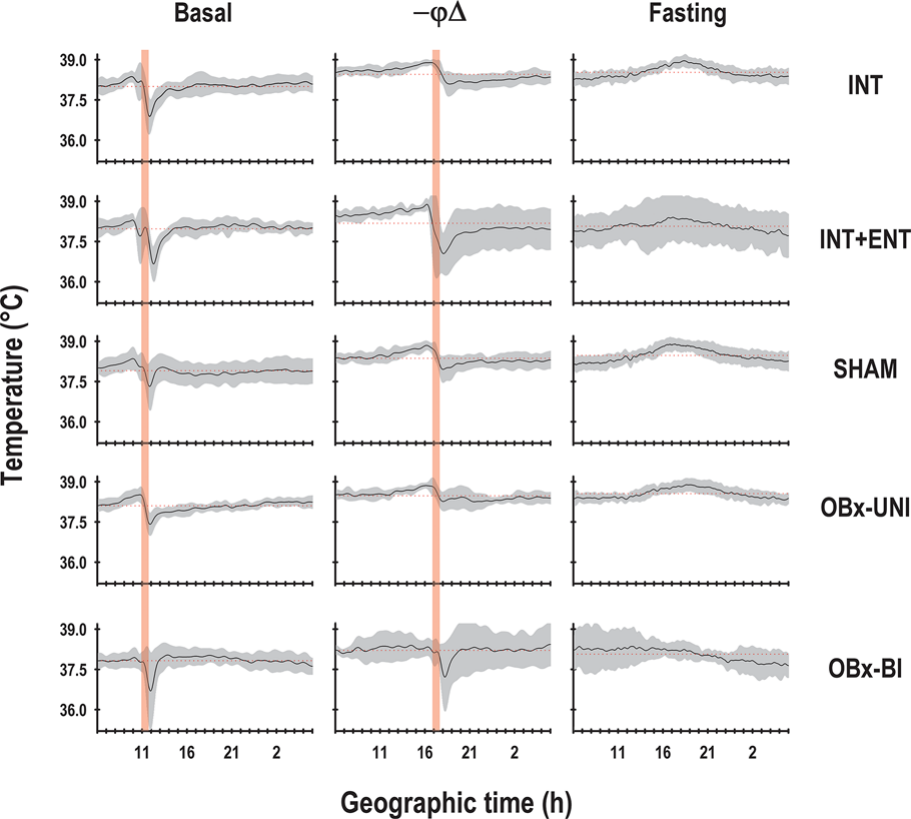
Mean temporal patterns of core body temperature. Average and standard error (SEM) of the diurnal profile of the temperature of intact rabbit pups (INT), intact pups fed by enteral gavage (INT+ENT), sham operated pups (SHAM), pups with unilateral lesions of the olfactory bulb (OBx-UNI) and pups with bilateral lesions of the olfactory bulb (OBx-BI). From postnatal day (P) 6–8, the pups were nursed or fed artificially every 24 h at 11:00 (indicated by the vertical red bar), from postnatal day 9–13 the feeding schedule was delayed 6 h (17:00), and from P14-15 the pups were maintained under fasting conditions. The dotted red line indicates the daily mean temperature.

With respect to the temporal pattern of the core body temperature in the basal segment (P6-8), the rabbits in the INT, INT+ENT, SHAM and OBx-UNI groups exhibited a characteristic diurnal rhythm (Fig 2) in which the core body temperature started to rise above the 24-h mean before the scheduled time of access to the lactating doe for nursing or artificial feeding. Following this episode, the average body temperature dropped almost immediately below the 24-h mean and remained at this low level for approximately 5 h (Fig 3). During P6-P8, Fourier analysis revealed that in 63–88% of cases, the 24-h component was present in the 3 most energetic components. In contrast, the newborn rabbits in the OBx-BI group exhibited an atypical temporal pattern of core body temperature from P6-8 that differed considerably from the other groups (Figs 2 and 3). All of the pups in the OBx-BI group exhibited temperatures near the 24-h temperature mean approximately 5 h before the scheduled time of access to the female or artificial feeding. Even during the experimental manipulation, the core body temperature remained near the 24-h mean. After manipulation, all of the animals exhibited a marked drop in temperature for approximately 1 to 3 h (Figs 2 and 3). In addition, Fourier analysis revealed that the 24-h component was evident in only 38% of cases in the OBx-BI rabbits during P6-P8.

In the second segment (P9-13), in which the nursing or artificial feeding schedule was delayed, the animals in the INT, INT+ENT, SHAM and OBx-UNI groups exhibited close similarities in expression of the entrained core body temperature rhythm. On the day of the shift in time of access to the doe for nursing or artificial feeding (P9), body temperature was maintained above the 24-h mean for approximately four hours after the previously scheduled nursing time. By P10, the pups started to show an increase in body temperature before 17:00, corresponding to the new nursing time, followed by an abrupt decline after nursing below the 24-h mean. By P11, the pups showed clear changes in body temperature in relation to the new nursing time (Figs 2 and 3). In contrast, the rabbits in the OBx-BI group exhibited an unstable temporal pattern during P9-13, with only a conspicuous decrease in temperature produced by the nursing or feeding events (Figs 2 and 3). Fourier analysis revealed that in 88–100% of cases, the 24-h component was evident in the 3 most energetic components in the rabbits in the INT, SHAM and OBx-UNI groups. In contrast, in the INT+ENT and OBx-BI groups, the 24-h component was only evident in 63% of cases.

In the last experimental segment (P14-15), in which the animals were maintained under fasting conditions, the results for the INT, INT+ENT, SHAM and OBx-UNI groups were very similar because the anticipatory rise in temperature occurred in relation to the new time of nursing and persisted during the two cycles in which the rabbits did not received maternal cyclical cues (Figs 2 and 3). In contrast, in the rabbits in the OBx-BI group, the anticipatory component was present around the original feeding schedule (Figs 2 and 3).

**Fig 4:**
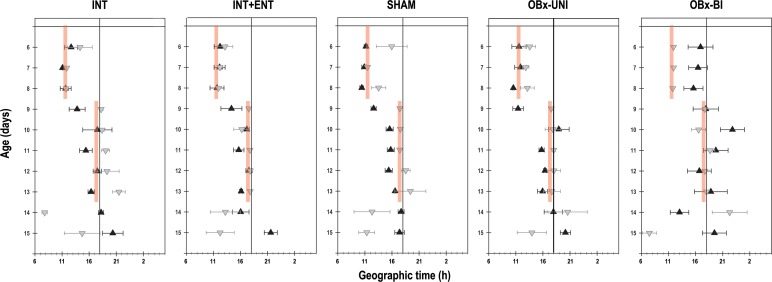
Daily phases of core body temperature. The daily acrophases (black triangles) and nadirs (gray triangles) of the diurnal pattern of core body temperature in intact rabbit pups (INT), intact pups fed by enteral gavage (INT+ENT), sham operated pups (SHAM), pups with unilateral lesions of the olfactory bulb (OBx-UNI) and pups with bilateral lesions of the olfactory bulb (OBx-BI). From postnatal day 6–8, the pups were nursed or fed artificially every 24 h at 11 (indicated by the vertical red bar), from postnatal day 9–13 the feeding schedule was delayed 6 h (17:00), and from P14-15 the pups were maintained under fasting conditions. The black line indicates one half of the cycle. Mean ± SEM.

Significant changes were evident in the acrophase of body temperature in pups in the experimental group and experimental segment (Group: F_4, 361_ = 4.1; *p* = 0.002; Segment: F_2, 361_ = 51.4; *p* = < 0.0001; Interaction: F_8, 361_ = 3.4; *p* = 0.0009). During the first segment of recording (P6-8), the acrophase of the diurnal rhythm of temperature in the rabbits in the INT, INT+ENT, SHAM and OBx-UNI groups occurred at 11:46 h ± 30 min, 11:59 h ± 38 min, 10:49 h ± 14 min and 11:18 h ± 31 min, respectively (Fig 4). In contrast, in the OBx-BI group, the acrophase was unstable, occurring at 16:18 h ± 65 min (Fig 4). After the 6-h phase delay of the scheduled time of access to the doe for nursing or artificial feeding (segment 2), the rabbits in the INT, INT+ENT, SHAM and OBx-UNI groups required approximately 2 cycles to show clear changes in body temperature in relation to the new feeding time; the acrophase of the diurnal rhythm of temperature occurred at 16:07 h ± 41 min, 15:58 h ± 32 min, 15:09 h ± 20 min and 15:41 h ± 36 min, respectively. In contrast, in the OBx-BI group, the acrophase of the temperature rhythm remained markedly unstable, occurring at 19:11 h ± 64 min (Fig 4). In the last experimental segment (P14-15), in which the animals were maintained under fasting conditions, the acrophase in the INT, INT+ENT, SHAM and OBx-UNI groups occurred at 19:17 h ± 59 min, 18:49 h ± 71 min, 17:32 h ± 30 min and 19:00 h ± 59 min, respectively. In the OBx-BI group, the acrophase of the temperature rhythm occurred earlier, at 16:14 h ± 95 min (Fig 4).

Significant changes were evident in the nadir of the body temperature of the pups in the experimental segment and interaction (Group: F_4, 383_ = 1.9; *p* = NS; Segment: F_2, 383_ = 59.9; *p* < 0.0001; Interaction: F_8, 383_ = 2.7; *p* = 0.007). During the first segment of recording (P6-8), the rabbits in all groups displayed a nadir of the diurnal rhythm of temperature between 1 h 30 min—2 h after the feeding time. After the 6-h phase delay of the scheduled time of access to the doe for nursing or artificial feeding (segment 2), the nadir shifted, occurring between 30 min—2 h after the new feeding time. Finally, in the third segment (P14-15), when the animals remained under fasting conditions, the nadir of the temperature rhythm became unstable and occurred between 5 h before– 15 min after the last scheduled time of access to the doe for nursing or artificial feeding (Fig 4).

**Fig 5:**
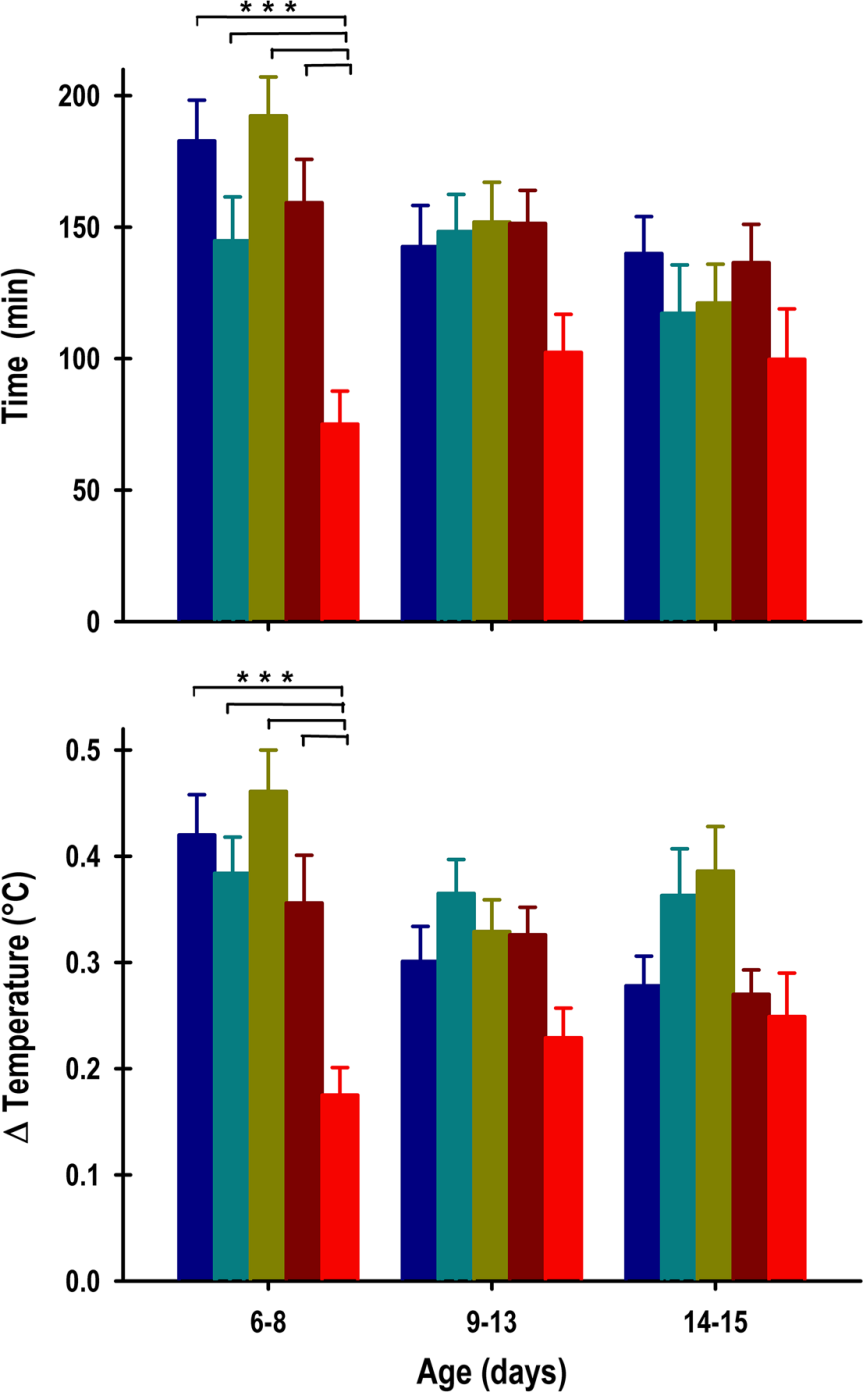
The anticipatory component of core body temperature. Graphics of the duration (top panel) and intensity (bottom panel) of the anticipation in core body temperature of intact rabbit pups (INT, dark blue bars), intact pups fed by enteral gavage (INT+ENT, dark cyan bars), sham operated pups (SHAM, dark green bars), pups with unilateral lesions of the olfactory bulb (OBx-UNI, dark red bars) and pups with bilateral lesions of the olfactory bulb (OBx-BI, red bars). From postnatal day 6–8, the pups were nursed or fed artificially every 24 h at 11:00, from postnatal day 9–13 the feeding schedule was delayed 6 h (17:00), and from P14-15 the pups were maintained under fasting conditions. Mean ± SEM. Fisher *** *p*<0.01.

The duration of the pups’ anticipation of nursing revealed significant changes in the experimental group (Group: F_4, 381_ = 7.3; *p* = < 0.0001; Segment: F_2, 381_ = 2.8; *p* = NS; Interaction: F_8, 381_ = 1.3; *p* = NS). In the first segment of recording (P6-8), the INT, INT+ENT, SHAM and OBx-UNI groups exhibited an anticipatory rise in temperature of 182 ± 16 min, 145 ± 17 min, 192 ± 15 min, and 159 ± 17 min, respectively (Fig 5, top panel). The pups in the OBx-BI group exhibited a significant decrease in the anticipatory rise in temperature of 75 ± 13 min compared with the remaining groups (Fig 5, top panel). After the 6-h phase delay of the scheduled time of access to the doe for nursing or artificial feeding (segment 2), the INT, INT+ENT, SHAM, OBx-UNI and OBx-BI groups exhibited a similar anticipatory rise in temperature, of 143 ± 16 min, 148 ± 14 min, 152 ± 15 min, 151 ± 13 min, and 102 ± 14 min, respectively (Fig 5, top panel). In the third segment (P14-15), when the animals remained under fasting conditions, the INT, INT+ENT, SHAM, OBx-UNI and OBx-BI groups exhibited a similar anticipatory rise in temperature of 140 ± 14 min, 117 ± 18 min, 121 ± 15 min, 137 ± 15 min, and 100 ± 19 min, respectively (Fig 5, top panel).

The intensity of the anticipatory rise in core body temperature revealed significant changes in the experimental group and segment (Group: F_4, 382_ = 10; *p* = < 0.0001; Segment: F_2, 382_ = 3.3; *p* = 0.03; Interaction: F_8, 382_ = 1.9; *p* = NS). During the first segment of recording (P6-8), similar temperature increases were observed during the anticipatory rise in the INT, INT+ENT, SHAM, and OBx-UNI groups of 0.42 ± 0.04°C, 0.38 ± 0.03°C, 0.46 ± 0.04°C, and 0.36 ± 0.05°C, respectively (Fig 5, bottom panel). In contrast, the pups in the OBx-BI group exhibited a significant decrement in the magnitude of the anticipatory rise (0.18 ± 0.03°C) compared with the remaining groups (Fig 5 bottom panel). After the 6-h phase delay of the scheduled time of access to the doe for nursing or artificial feeding (segment 2), the INT, INT+ENT, SHAM, and OBx-UNI groups exhibited a similar increase in temperature of 0.3 ± 0.03°C, 0.37 ± 0.03°C, 0.33 ± 0.03°C, and 0.33 ± 0.03°C, respectively (Fig 5, bottom panel). The OBx-BI pups continued to exhibit the same tendency, with a conspicuous decrease in the magnitude of the anticipatory rise (0.23± 0.02°C). In the third segment (P13-15), when the animals remained under fasting conditions, the INT, INT+ENT, SHAM, OBx-UNI and OBx-BI groups exhibited a similar temperature increase of 0.28 ± 0.03°C, 0.36 ± 0.04°C, 0.39 ± 0.04°C, 0.27 ± 0.02°C, and 0.25 ± 0.04°C, respectively (Fig 5, bottom panel).

It is noteworthy that during all experiments, the duration and magnitude of the anticipatory component tended to be inferior in the pups in the OBx-BI group compared with the remaining groups.

### Gross Locomotor Activity

**Fig 6:**
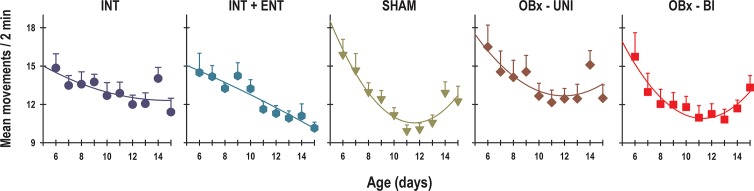
Daily average gross locomotor activity. The mean activity from postnatal day 6–15 of intact rabbit pups (INT, dark blue circles), intact pups fed by enteral gavage (INT+ENT, dark cyan hexagons), sham operated pups (SHAM, dark green triangles), pups with lesions of the olfactory bulb (OBx-UNI, dark red diamonds), and pups with bilateral lesions of the olfactory bulb (OBx-BI, red squares). Mean ± SEM, r^2^ = 0.97.

A 2-way ANOVA revealed significant effects of the experimental manipulation and age on the average locomotor activity of newborn rabbits (Group: F_4, 344_ = 3.9; *p* = 0.003; Age: F_9, 344_ = 8.9; *p* = 0.0001; Interaction: F_36, 344_ = 0.8; *p* = NS). The daily average activity of the rabbit pups decreased significantly in a manner consistent with age (Fig 6). The mean activity of the INT pups on P6 was 14.8 ± 1.1 movements per sample (mps, mean counts per 2 min), which decreased gradually to 11.4 ± 1.1 mps on P15. Similarly, the newborn rabbits in the INT+ENT, SHAM, OBx-UNI and OBx-BI groups exhibited an initial activity on P6 of 14.5 ± 1.2 mps, 15.9 ± 1.2 mps, 16.5 ± 1.7 mps and 15.7 ± 1.8 mps, respectively. At the end of the experiment on P15, the average activity was 10.2 ± 0.5 mps, 12.3 ± 1.2 mps, 12.5 ± 1 mps and 13.3 ± 0.9 mps, respectively (Fig 6).

**Fig 7:**
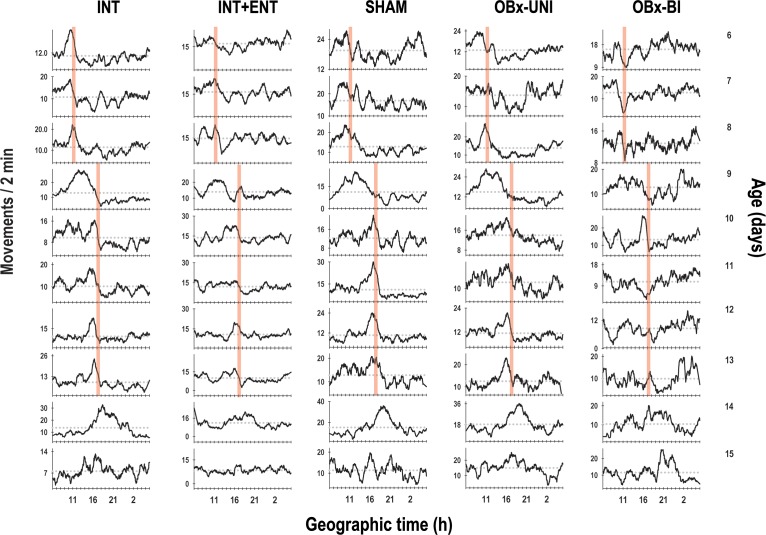
Temporal patterns of gross locomotor activity. Representative daily profiles of activity in newborn rabbits as measured using biotelemetry, in intact pups (INT), intact pups fed by enteral gavage (INT+ENT), sham operated pups (SHAM), pups with unilateral lesions of the olfactory bulb (OBx-UNI) and pups with bilateral lesions of the olfactory bulb (OBx-BI). From postnatal day 6–8, the pups were nursed or fed artificially every 24 h at 11:00 (indicated by the vertical red bar), from postnatal day 9–13 the feeding schedule was delayed 6 h (17:00), and from P14-15 the pups were maintained under fasting conditions (see text for details). The red dotted line indicates the daily mean activity.

**Fig 8:**
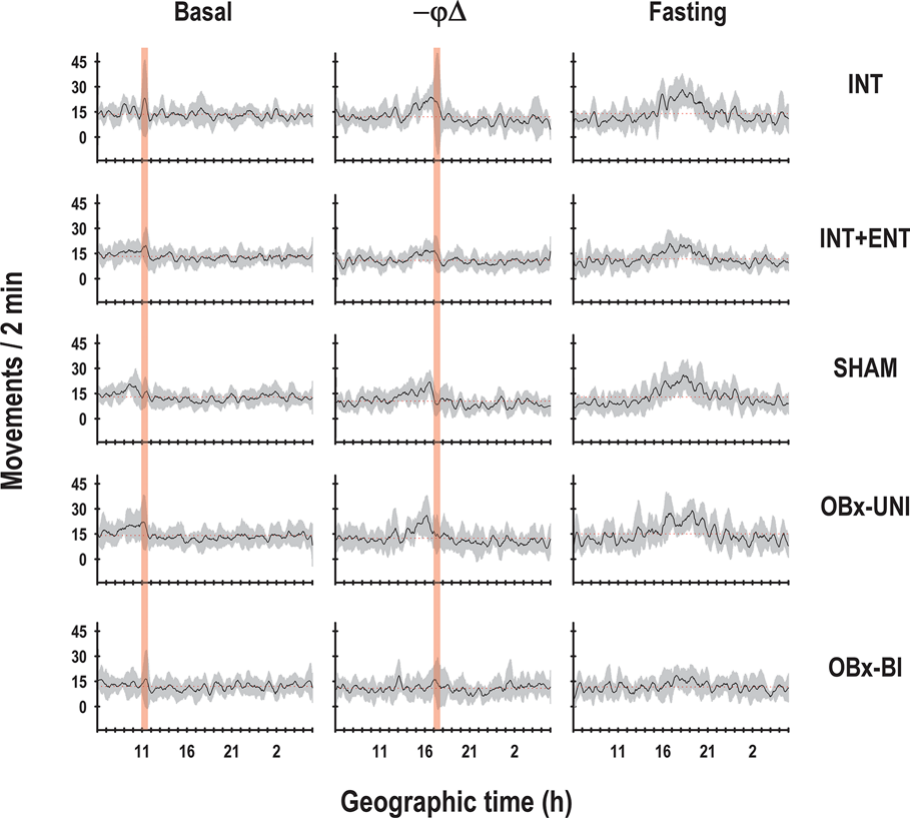
Mean temporal patterns of gross locomotor activity. Average and standard error (SEM) of the diurnal profile of activity in intact rabbit pups (INT), intact pups fed by enteral gavage (INT+ENT), sham operated pups (SHAM), pups with unilateral lesions of the olfactory bulb (OBx-UNI) and pups with bilateral lesions of the olfactory bulb (OBx-BI). From postnatal day (P) 6–8, the pups were nursed or fed artificially every 24 h at 11:00 (indicated by the vertical red bar), from postnatal day 9–13 the feeding schedule was delayed 6 h (17:00), and from P14-15 the pups were maintained under fasting conditions. The red dotted line indicates the daily mean activity.

The temporal pattern of the gross locomotor activity in the basal segment (P6-8) revealed a characteristic diurnal rhythm in the rabbits in the INT, INT+ENT, SHAM and OBx-UNI groups (Fig 7), in which the activity started to rise above the 24-h mean before the scheduled time of access to the lactating doe for nursing or artificial feeding. Following this episode, the average activity dropped almost immediately below the 24-h mean and remained at this low level for approximately 1–2 h (Figs 7 and 8). During P6-P8, Fourier analysis revealed that in 75–100% of cases, the 24-h component was evident in the 3 most energetic components. In contrast, newborn rabbits in the OBx-BI group did not exhibit a fully consolidated diurnal pattern of activity from P6-8, and an isolated case showed the anticipatory increase in activity before the scheduled time of access to the female or artificially feeding (Figs 7 and 8). In addition, after manipulation, all of the animals in the OBx-BI group displayed a marked decline in activity for approximately 1 h (Figs 7 and 8). Fourier analysis revealed that the 24-h component was evident in only 75% of cases in the OBx-BI rabbits from P6-P8, and in one case, this frequency was not evident.

In the second segment (P9-13), in which the nursing or artificial feeding schedule was delayed, animals in the INT, INT+ENT, SHAM and OBx-UNI groups exhibited close similarities in expression of the entrained locomotor activity rhythm. On the day of the shift in time of access to the doe for nursing or artificial feeding (P9), the activity was maintained above the 24-h mean for approximately five hours after the previously scheduled nursing or feeding time. By P10, the pups started to show an increase in activity at approximately 17:00 that corresponded to the new nursing or feeding time, followed by an abrupt decline after nursing below the 24-h mean. By P11, the pups showed clear changes in locomotor activity in relation to the new time of nursing or feeding (Figs 7 and 8). The rabbits in the OBx-BI group displayed conspicuous deficiencies in phase control of the activity rhythm, and on the day of the shift in time of access to the doe and artificial feeding, four of eight pups maintained the level of activity above the 24-h mean for approximately five hours after the previously scheduled feeding time. In the subsequent cycles, the anticipatory rise to feeding was again evident in isolated cycles, and up to P12 or 13, some pups in the OBx-BI group displayed clear changes in locomotor activity in relation to the new feeding time (Figs 7 and 8). Fourier analysis revealed that in 88–100% of cases, the 24-h component was evident in the 3 most energetic components in rabbits in the INT, SHAM and OBx-UNI groups. In contrast, in the INT+ENT and OBx-BI groups, the 24-h component was evident in only 50% of cases, and again in one animal in the OBx-BI group, this frequency was not apparent.

In the last experimental segment (P14-15), in which the animals were maintained under fasting conditions, very similar outcomes were observed in the INT, INT+ENT, SHAM and OBx-UNI groups because the anticipatory rise at the behavioral level occurred in relation to the last episode of nursing and persisted for two cycles in which the rabbits did not received maternal cyclical cues (Figs 7 and 8). The OBx-UNI group also exhibited an anticipatory rise close to the last feeding schedule; nevertheless, in the second cycle, a decrement in the amplitude of the anticipatory rise was observed (Figs 7 and 8). In the rabbits in the OBx-BI group, the persistence of the anticipatory component was unstable, and in the most cases was not evident, in the second cycle of the fasting condition (Figs 7 and 8). Fourier analysis revealed that in 88–100% of cases, the 24-h component was evident in the 3 most energetic components in the rabbits in the INT, INT+ENT, SHAM and OBx-UNI groups. In contrast, in the OBx-BI group, the 24-h component was evident in only 50% of cases, and again in one case, this frequency was not apparent.

**Fig 9:**
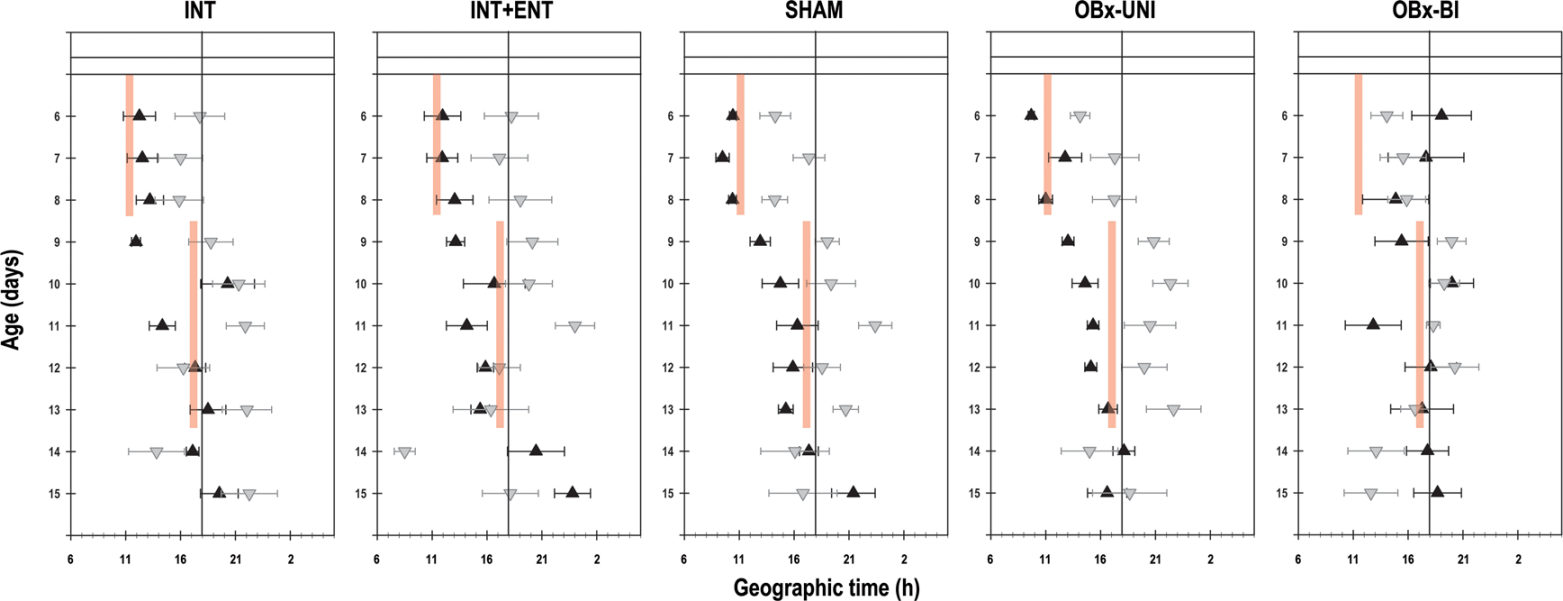
Daily phases of gross locomotor activity. The daily acrophases (black triangles) and nadirs (gray triangles) of the diurnal pattern of gross locomotor activity in intact rabbit pups (INT), intact pups fed by enteral gavage (INT+ENT), sham operated pups (SHAM), pups with unilateral lesions of the olfactory bulb (OBx-UNI) and pups with bilateral lesions of the olfactory bulb (OBx-BI). From postnatal day 6–8, the pups were nursed or fed artificially every 24 h at 11:00 (indicated by the vertical red bar), from postnatal day 9–13 the feeding schedule was delayed 6 h (17:00), and from P14-15 the pups were maintained under fasting conditions. The black line indicates one half of the cycle. Mean ± SEM.

Regarding the acrophases of the locomotor activity of the pups, significant changes were evident in the experimental group, segment and their interaction (Group: F_4, 348_ = 3.9; *p* = 0.003; Segment: F_2, 348_ = 47.4; *p* = < 0.0001; Interaction: F_8, 348_ = 3.9; *p* = 0.0002). During the first segment of recording (P6-8), the acrophase of the diurnal rhythm of activity in the rabbits in the INT, INT+ENT, SHAM and OBx-UNI groups occurred at 10:47 h ± 23 min, 10:29 h ± 53 min, 10:06 h ± 27 min and 10:59 h ± 57 min, respectively (Fig 9). In contrast, the acrophase was unstable in the OBx-BI group and occurred at 17:11 h ± 134 min (Fig 9). After the 6-h phase delay of the scheduled time of access to the doe for nursing or artificial feeding (P9-13), the rabbits in the INT, INT+ENT, SHAM and OBx-UNI groups required approximately 2 cycles to show clear changes in activity in relation to the new feeding time; the acrophase occurred at 16:40 h ± 83 min, 15:02 h ± 72 min, 14:01 h ± 67 min and 14:43 h ± 35 min, respectively. In contrast, in the OBx-BI group, the acrophase continued to be markedly unstable, remaining close to the previous phase observed in the first segment and to the new feeding schedule (16:42 h ± 70 min; Fig 9). In the last experimental segment (P14-15), in which the animals were maintained under fasting conditions, the acrophase of the activity rhythm in the INT, INT+ENT, SHAM and OBx-UNI groups occurred at 18:19 h ± 93 min, 22:08 h ± 154 min, 19:22 h ± 77 min and 17:22 h ± 61 min, respectively. In the OBx-BI group, the acrophase of the activity rhythm remained close to the previous phase observed in the first and second segment (18:14 h ± 100 min; Fig 9).

Significant changes were evident in the nadir of locomotor activity in the experimental segment (Group: F_4, 383_ = 2; *p* = NS; Segment: F_2, 383_ = 22.2; *p* = < 0.0001; Interaction: F_8, 383_ = 1.2; *p* = NS). During the first segment of recording (P6-8), the nadir of the diurnal rhythm of activity occurred between 5–7 h after the feeding time in all groups. After the 6-h phase delay of the scheduled time of access to the doe for nursing or artificial feeding (segment 2), the nadir of activity also shifted to between 4–6 h after the new feeding time. Finally, in the third segment (P14-15), during which the animals remained under fasting conditions, the nadir of the behavioral rhythm was unstable and occurred between 4 h before—1 h after the last scheduled time of access to the doe for nursing or artificial feeding (Fig 9).

**Fig 10:**
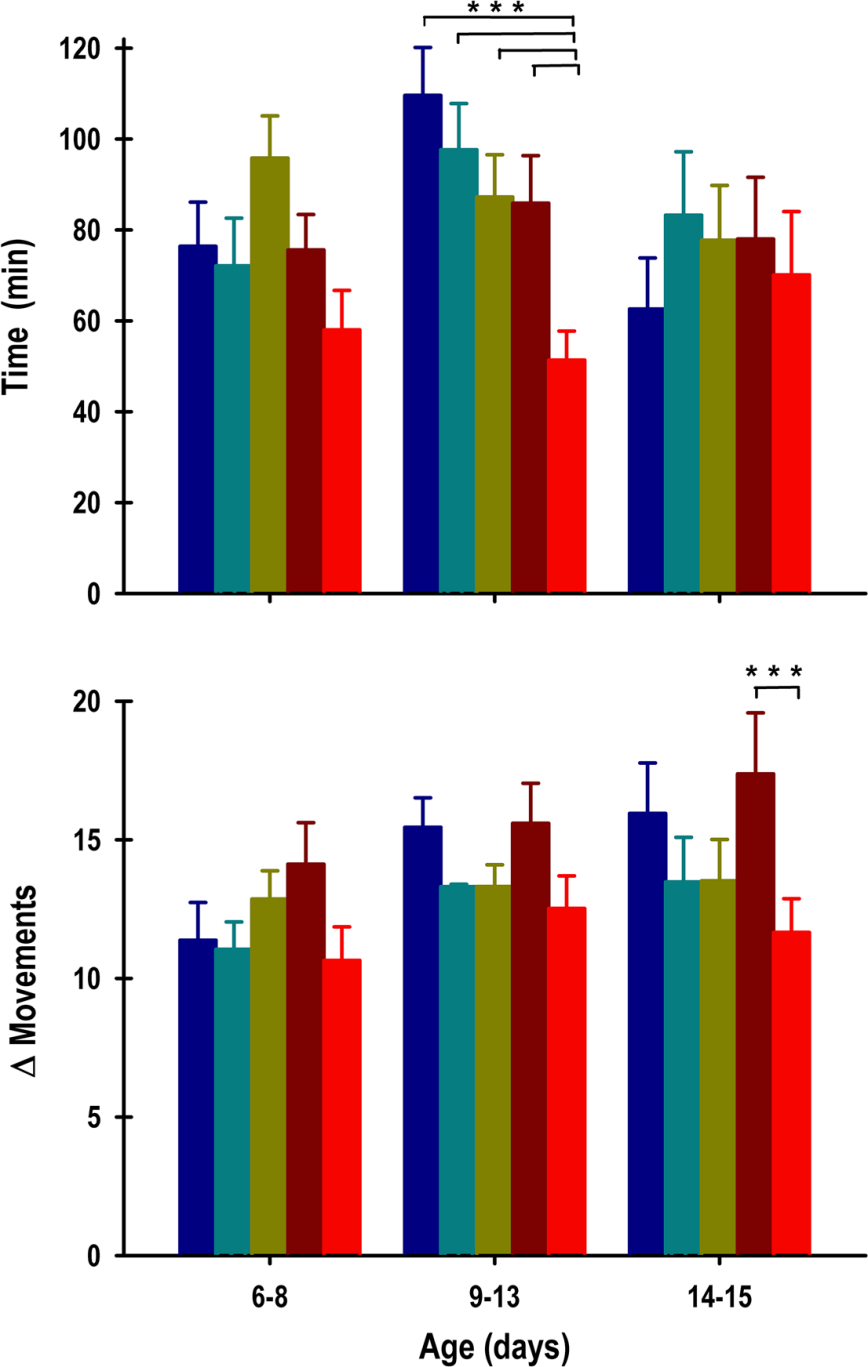
The anticipatory component of gross locomotor activity. Graphics of the duration (top panel) and intensity (bottom panel) of anticipation in gross locomotor activity of intact rabbit pups (INT, dark blue bars), intact pups fed by enteral gavage (INT+ENT, dark cyan bars), sham operated pups (SHAM, dark green bars), pups with unilateral lesions of the olfactory bulb (OBx-UNI, dark red bars) and pups with bilateral lesions of the olfactory bulb (OBx-BI, red bars). From postnatal day 6–8, the pups were nursed or fed artificially every 24 h at 11:00, from postnatal day 9–13 the feeding schedule was delayed 6 h (17:00), and from P14-15 the pups were maintained under fasting conditions. Mean ± SEM. Fisher *** *p*<0.01.

The experimental group displayed significant changes in the duration of the pups’ anticipation of nursing (Group: F_4, 383_ = 2.8; *p* = 0.02; Segment: F_2, 383_ = 2.1; *p* = NS; Interaction: F_8, 383_ = 1.6; *p* = NS). During the first segment of recording (P6-8), the INT, INT+ENT, SHAM, and OBx-UNI groups exhibited a similar tendency in the anticipatory rise in activity of 76.8 ± 10 min, 72 ± 11 min, 96 ± 9 min, and 76 ± 8 min, respectively (Fig 10, top panel). In the OBx-BI group, the anticipatory rise was 58 ± 9 min (Fig 10, top panel). After the 6-h phase delay of the scheduled time of access to the doe for nursing or artificial feeding (segment 2), the INT, INT+ENT, SHAM and OBx-UNI groups exhibited a similar duration of the anticipatory rise in activity of 110 ± 11 min, 98 ± 10 min, 87 ± 9 min, and 86 ± 10 min, respectively (Fig 10, top panel). In contrast, the pups in the OBx-BI group exhibited a significant decrement in the duration of the anticipatory rise that persisted 51 ± 6 min compared with the remaining groups (Fig 10, top panel). In the third segment (P14-15), in which the animals remained under fasting conditions, the INT, INT+ENT, SHAM, OBx-UNI and OBx-BI groups exhibited a similar duration in the anticipatory rise in activity of 62.6 ± 11 min, 83 ± 14 min, 78 ± 12 min, 78 ± 14 min, and 70 ± 14 min, respectively (Fig 10, top panel).

Significant changes in the intensity of the anticipatory rise in gross locomotor activity were observed in the experimental group and segment (Group: F_4, 380_ = 3.7; *p* = 0.005; Segment: F_2, 380_ = 4.4; *p* = 0.01; Interaction: F_8, 380_ = 0.5; *p* = NS). During the first segment of recording (P6-8), similar increases in activity were observed during the anticipatory rise in the INT, INT+ENT, SHAM, OBx-UNI and OBx-BI groups (11.4 ± 1.4 mps, 11.1 ± 1 mps, 12.9 ± 1 mps, 14.1 ± 1.5 mps, and 10.6 ± 1.2 mps, respectively; Fig 10, bottom panel). After the 6-h phase delay of the scheduled time of access to the doe for nursing or artificial feeding (segment 2), the INT, INT+ENT, SHAM, OBx-UNI, and OBx-BI groups exhibited a similar increase in activity of 15.5 ± 1.1 mps, 13.3 ± 0.9 mps, 13.3 ± 0.8 mps, and 15.6 ± 1.4 mps, and 12.5± 1.2 mps, respectively (Fig 10, bottom panel). In the third segment (P13-15), during which the animals remained under fasting conditions, the INT, INT+ENT, SHAM, and OBx-UNI groups exhibited a similar increase in locomotor activity of 15.9 ± 1.8 mps, 13.5 ± 1.6 mps, 13.5 ± 1.5 mps, and 17.4 ± 2.3 mps, respectively (Fig 10, bottom panel). In contrast, the pups in the OBx-BI group exhibited a significant decrement in the magnitude of the anticipatory rise (11.7 ± 1.2 mps) compared with those in the OBx-UNI group (Fig 10, bottom panel).

## Discussion

In this study, we report that total removal of the main olfactory bulb in newborn rabbits has significant effects on the consolidation, phase control and maintenance of the 24-h pattern of core body temperature and, to some extent, on the expression of the gross locomotor activity rhythm. This experimental evidence indicates that the olfactory bulb modulates circadian rhythmicity, in particular during early non-photic stages of development.

The daily mean body temperature and locomotor activity underwent significant changes with growth in rabbit pups, consistent with previous reports [23, 24, 44]. The core body temperature exhibited a gradual increase, whereas the levels of locomotor activity decreased with the age of the rabbits. Nevertheless, it is noteworthy that newborn rabbits with total removal of the MOB exhibited hypothermia at the end of the experiment. A similar effect has been described in rabbit pups raised without maternal volatile chemical cues [21]. It is known that olfactory stimulation modulates the autonomic nervous system [45, 46], and it is possible that the absence of maternal olfactory cues during the early stages of development could alter the thermoregulatory ability of newborn rabbits by modulating the function of the autonomic nervous system.

Consistent with previous studies [24–25, 47], newborn rabbits in the INT, INT+ENT, and SHAM groups exhibited a characteristic diurnal pattern in core temperature and locomotor activity, with a conspicuous anticipatory rise in temperature and activity prior to the time of access to the doe for nursing or artificial feeding, followed by a noticeable diminution in both parameters during the postprandial period. In addition, the pups in the INT, INT+ENT, and SHAM groups exhibited a clear and stable phase relationship between the time of nursing or olfactory stimulation followed by artificial feeding and maximal temperature and activity, even after the phase delay of the schedule. In both parameters, the 24-h fluctuation persisted even when nursing or feeding was omitted.

Despite unilateral removal of the MOB, the olfactory capacity of rabbit pups was not compromised because the pups in the OBx-UNI were able to orient themselves to the lactating female and to nurse efficiently during all experiments, in agreement with previous studies [48]. In addition, expression of the diurnal and circadian patterns of temperature and activity was unaffected. It is possible that the MOB comprises a network of self-sustained oscillating neurons, similarly to the hypothalamic master circadian pacemaker, in which every neuron shares the same molecular clockwork and is capable of oscillating independently (review in [49]). Only when the lesion affects at least 80% of the nuclei is total arrhythmicity produced at the behavioral level. This functional redundancy is also apparent in the MOB, such that partial or unilateral lesions do not eliminate the circadian organization.

Because total removal of the olfactory bulb in rabbit pups eliminates the stereotypical pattern of nipple searching behavior, as well as circadian regulation of the core body temperature and, to some extent, the gross locomotor rhythm, olfactory function was clearly disrupted. The most conspicuous effects observed in pups with total lesions of the MOB were an atypical temporal pattern of body temperature characterized by a diminution in the anticipatory component, poor phase control and attenuation of the rhythm under constant conditions. The decrement in the duration and intensity of the circadian-regulated anticipatory component in OBx-BI pups was mainly evident during the pre-visual stages of development, at which time the circadian system undergoes a major non-photic modulation [29, 32].

Total removal of both olfactory bulbs also affected the expression of the locomotor activity pattern in rabbits. This result was similar to findings reported using adult rodents and primates, in which bilateral elimination of the MOB markedly modified the period, phase control and overall architecture of the activity rhythm [16, 19, 50–52], supporting a modulatory role of the olfactory system in circadian organization, even in the presence of the light-entrainable pacemaker.

During the first 10 days of life, olfaction plays a prominent role in the survival of rabbit pups because it is the more developed sensorial system [53]. It is well known that newborn rabbits naturally reside in subterranean burrows under constant dark conditions [31], their eyelids are closed and the visual system is not yet functional [54, 55]. In addition, circadian regulation does not appear to depend solely on the light-entrainable hypothalamic circadian pacemaker because the molecular clockwork is not fully mature at this age and lesions of the SCN do not eliminate the core body temperature rhythm [29, 36]. It is worth exploring the effect of the simultaneous elimination of the MOB and SCN on temporal regulation in mammals after birth.

Our current data demonstrate that circadian regulation was affected in rabbit pups bearing bilateral lesions of the MOB. Previous findings have indicated that during early development, circadian organization can be achieved by the olfactory system. For example, the molecular clockwork matures earlier in the MOB, even prior to the SCN, such that the clock genes *Per1*, *Bmal1* and *Cry1* exhibit a consolidated rhythmicity in the MOB as early as postnatal day 7. In addition, daily exposure to maternal chemical cues such as mammary pheromone can modulate the rhythmic expression at behavioral and physiological levels [21], as well as the expression of core clock proteins in the MOB, in a time-dependent manner [32]. Thus, it is possible that during early stages of development when the olfactory bulb is functional and has strong adaptive characteristics, it also participates directly in the modulation of newborn circadian rhythmicity as the SCN matures and photic pathways and signals increase the saliency for rabbits, promoting the re-organization of the circadian system. Nevertheless, it remains crucial to assess the effect of complete removal of the MOB on the coupling of the peripheral and central oscillators.

It is important to note that in the INT+ENT pups, daily exposure to a female impregnated with the mammary pheromone 2MB-2 was capable of entraining patterns of temperature and activity. This result is consistent with that of a previous study in which daily exposure to fresh maternal milk or 2-MB2 regulated diurnal rhythmicity in rabbit pups [21]. These findings support the hypothesis that maternal chemical cues have a prominent role as non-photic modulators and that the olfactory system participates in the generation of circadian rhythmicity during early stages of development.

The current findings indicate that the expression of circadian rhythmicity at behavioral and physiological levels during early stages of rabbit development largely depends on the integrity of the main olfactory bulb.

## Supporting Information

S1 Fig Representative histological microphotographs of the olfactory bulb. Images of the olfactory bulb of intact rabbit pups (INT), intact pups fed by enteral gavage (INT+ENT), sham operated pups (SHAM), pups with unilateral lesions of the olfactory bulb (OBx-UNI) and pups with bilateral lesions of the olfactory bulb (OBx-BI).
(TIF)

S2 Fig Daily average body weight. The mean weight from postnatal day 0–13 of intact rabbit pups (INT, open circles), intact pups fed by enteral gavage (INT+ENT, open hexagons), sham operated pups (SHAM, light gray triangles), pups with unilateral lesions of the olfactory bulb (OBx-UNI, dark gray diamonds), and pups with bilateral lesions of the olfactory bulb (OBx-BI, black squares). Mean ± SEM, r^2^ = 0.95. ❖, ♓ and ⌘ indicates a significant difference (p<0.05) *vs*. INT, SHAM and OBx-UNI.
(TIF)

S1 Text Core body temperature data set. Results of two-way ANOVA and Scheffe post-hoc test, of core body temperature daily average, phases, and the duration and intensity of the anticipatory component, of intact rabbit pups, intact pups fed by enteral gavage, sham operated, with unilateral lesions of the olfactory bulb, and with bilateral lesions of the olfactory bulb.
(PDF)

S2 Text Locomotor activity data set. Results of two-way ANOVA and Scheffe post-hoc test, of locomotor activity daily average, phases, and the duration and intensity of the anticipatory component, of intact rabbit pups, intact pups fed by enteral gavage, sham operated, with unilateral lesions of the olfactory bulb, and with bilateral lesions of the olfactory bulb.
(PDF)
