# Supplementary figures and images for: Participation of the Olfactory Bulb in Circadian Organization during Early Postnatal Life in Rabbits
Source: PLoS One. 2016 Jun 15;11(6):e0156539. doi: 10.1371/journal.pone.0156539 (PMC4909232; doi:10.1371/journal.pone.0156539)

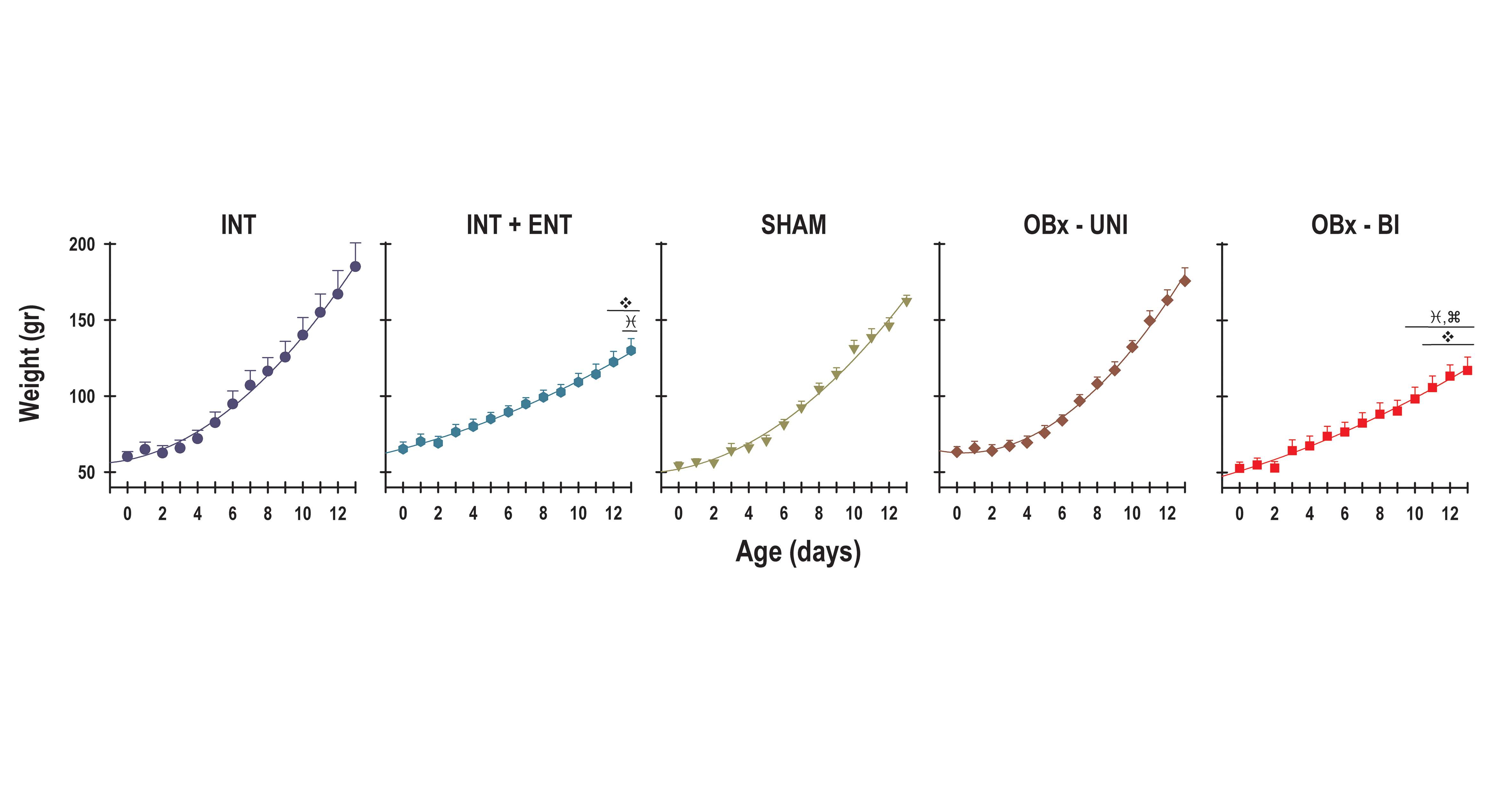

Supplement: S2 Fig — The mean weight from postnatal day 0–13 of intact rabbit pups (INT, open circles), intact pups fed by enteral gavage (INT+ENT, open hexagons), sham operated pups (SHAM, light gray triangles), pups with unilateral lesions of the olfactory bulb (OBx-UNI, dark gray diamonds), and pups with bilateral lesions of the olfactory bulb (OBx-BI, black squares). Mean ± SEM, r2 = 0.95. ❖, ♓ and ⌘ indicates a significant difference (p<0.05) vs. INT, SHAM and OBx-UNI. (TIF) [file pone.0156539.s002.tif]
