# Supplementary material for: Participation of the Olfactory Bulb in Circadian Organization during Early Postnatal Life in Rabbits
Source: PLoS One. 2016 Jun 15;11(6):e0156539. doi: 10.1371/journal.pone.0156539 (PMC4909232; doi:10.1371/journal.pone.0156539)
Supplement: S1 Text — Results of two-way ANOVA and Scheffe post-hoc test, of core body temperature daily average, phases, and the duration and intensity of the anticipatory component, of intact rabbit pups, intact pups fed by enteral gavage, sham operated, with unilateral lesions of the olfactory bulb, and with bilateral lesions of the olfactory bulb. (PDF) [file pone.0156539.s003.pdf]

Core Body Temperature
Daily Average

ANOVA Table for Column 7

	DF	Sum of Squares	Mean Square	F-Value	P-Value
Column 4	4	4.018	1.005	11.087	<.0001
Column 6	9	11.362	1.262	13.934	<.0001
Column 4 * Column 6	36	4.325	.120	1.326	.1059
Residual	348	31.531	.091		

6724 cases were omitted due to missing values.

Means Table for Column 7
Effect: Column 4

	Count	Mean	Std. Dev.	Std. Err.
BI	79	38.171	.442	.050
INT	80	38.401	.275	.031
INT+ENT	80	38.166	.452	.051
SHAM	79	38.300	.237	.027
UNI	80	38.389	.261	.029

6724 cases were omitted due to missing values.

Interaction Bar Plot for Column 7
Effect: Column 4
Error Bars: 95% Confidence Interval

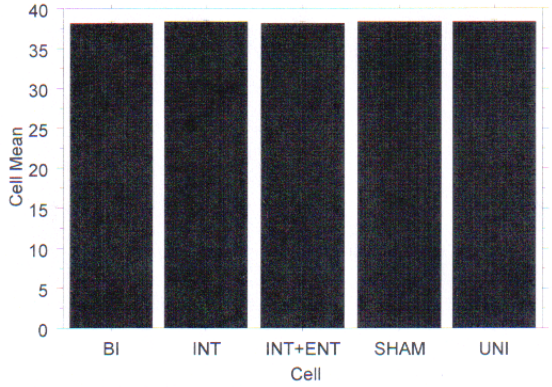

6724 cases were omitted due to missing values.

Bonferroni/Dunn for Column 7
Effect: Column 4
Significance Level: 5 %

	Mean Diff.	Crit. Diff	P-Value	
BI, INT	-.230	.135	<.0001	S
BI, INT+ENT	.005	.135	.9168	
BI, SHAM	-.129	.135	.0073	
BI, UNI	-.218	.135	<.0001	S
INT, INT+ENT	.235	.134	<.0001	S
INT, SHAM	.101	.135	.0360	
INT, UNI	.012	.134	.7985	
INT+ENT, SH...	-.134	.135	.0052	
INT+ENT, UNI	-.223	.134	<.0001	S
SHAM, UNI	-.088	.135	.0651	

Comparisons in this table are not significant unless the corresponding p-value is less than .005.
6724 cases were omitted due to missing values.

Means Table for Column 7
Effect: Column 4 * Column 6

	Count	Mean	Std. Dev.	Std. Err.
BI, P10	8	38.191	.101	.036
BI, P11	8	38.393	.380	.134
BI, P12	8	38.528	.391	.138
BI, P13	7	38.590	.650	.246
BI, P14	8	38.416	.522	.185
BI, P15	8	37.743	.459	.162
BI, P6	8	37.955	.186	.066
BI, P7	8	38.068	.173	.061
BI, P8	8	38.053	.188	.066
BI, P9	8	37.825	.232	.082
INT, P10	8	38.441	.255	.090
INT, P11	8	38.553	.271	.096
INT, P12	8	38.578	.185	.066
INT, P13	8	38.514	.145	.051
INT, P14	8	38.595	.189	.067
INT, P15	8	38.415	.257	.091
INT, P6	8	38.127	.215	.076
INT, P7	8	38.166	.187	.066
INT, P8	8	38.259	.324	.114
INT, P9	8	38.360	.278	.098
INT+ENT, P...	8	38.412	.171	.061
INT+ENT, P...	8	38.368	.225	.080
INT+ENT, P...	8	38.463	.233	.082
INT+ENT, P...	8	38.187	.722	.255
INT+ENT, P...	8	38.118	.945	.334
INT+ENT, P...	8	37.964	.497	.176
INT+ENT, P6	8	37.968	.214	.076
INT+ENT, P7	8	38.018	.120	.042
INT+ENT, P8	8	38.005	.173	.061
INT+ENT, P9	8	38.157	.231	.082
SHAM, P10	8	38.315	.218	.077
SHAM, P11	8	38.331	.188	.066
SHAM, P12	8	38.357	.127	.045
SHAM, P13	7	38.449	.149	.056
SHAM, P14	8	38.583	.211	.074
SHAM, P15	8	38.254	.305	.108
SHAM, P6	8	38.162	.173	.061
SHAM, P7	8	38.162	.145	.051
SHAM, P8	8	38.164	.234	.083
SHAM, P9	8	38.246	.275	.097
UNI, P10	8	38.394	.105	.037
UNI, P11	8	38.517	.133	.047
UNI, P12	8	38.606	.102	.036
UNI, P13	8	38.651	.155	.055
UNI, P14	8	38.668	.143	.051
UNI, P15	8	38.396	.192	.068
UNI, P6	8	38.039	.207	.073
UNI, P7	8	38.124	.218	.077
UNI, P8	8	38.226	.181	.064
UNI, P9	8	38.266	.139	.049

6724 cases were omitted due to missing values.

Core Body Temperature Achrophases

ANOVA Table for Column 7

	DF	Sum of Squares	Mean Square	F-Value	P-Value
Column 2	4	978075.729	244518.932	4.142	.0027
Column 5	2	6071482.197	3035741.099	51.425	<.0001
Column 2 * Column 5	8	1610821.880	201352.735	3.411	.0009
Residual	361	21310523.342	59031.921		

488 cases were omitted due to missing values.

Means Table for Column 7
Effect: Column 2

	Count	Mean	Std. Dev.	Std. Err.
BI	79	702.633	378.862	42.625
INT	76	578.263	275.834	31.640
INT+ENT	68	575.029	252.928	30.672
SHAM	78	501.744	182.968	20.717
UNI	75	557.387	262.654	30.329

488 cases were omitted due to missing values.

Interaction Bar Plot for Column 7
Effect: Column 2
Error Bars: 95% Confidence Interval

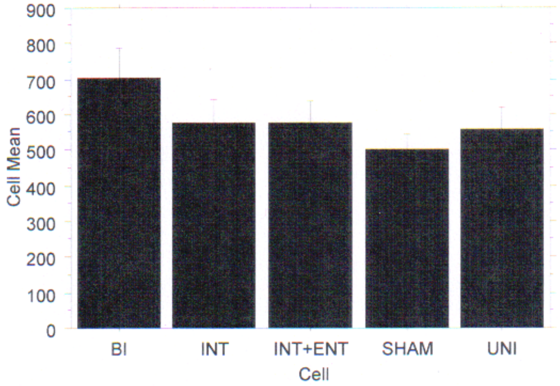

488 cases were omitted due to missing values.

Bonferroni/Dunn for Column 7
Effect: Column 2
Significance Level: 5 %

	Mean Diff.	Crit. Diff	P-Value	
BI, INT	124.370	110.259	.0016	S
BI, INT+ENT	127.603	113.517	.0016	S
BI, SHAM	200.889	109.536	<.0001	S
BI, UNI	145.246	110.633	.0002	S
INT, INT+ENT	3.234	114.548	.9365	
INT, SHAM	76.520	110.605	.0515	
INT, UNI	20.876	111.691	.5979	
INT+ENT, SH...	73.286	113.853	.0699	
INT+ENT, UNI	17.643	114.908	.6648	
SHAM, UNI	-55.643	110.978	.1576	

Comparisons in this table are not significant unless the corresponding p-value is less than .005.
488 cases were omitted due to missing values.

Means Table for Column 7
Effect: Column 2 * Column 5

	Count	Mean	Std. Dev.	Std. Err.
BI, S1	24	618.500	317.615	64.833
BI, S2	39	790.615	400.941	64.202
BI, S3	16	614.375	380.401	95.100
INT, S1	20	346.000	133.667	29.889
INT, S2	40	606.950	261.004	41.268
INT, S3	16	796.875	235.731	58.933
INT+ENT, S1	18	359.111	162.963	38.411
INT+ENT, S2	34	598.059	187.191	32.103
INT+ENT, S3	16	769.000	285.093	71.273
SHAM, S1	23	289.391	64.929	13.539
SHAM, S2	39	548.769	121.826	19.508
SHAM, S3	16	692.375	120.052	30.013
UNI, S1	19	318.000	134.224	30.793
UNI, S2	40	580.950	227.210	35.925
UNI, S3	16	782.750	237.171	59.293

488 cases were omitted due to missing values.

Core Body Temperature Nadir

ANOVA Table for Column 17

	DF	Sum of Squares	Mean Square	F-Value	P-Value
Column 11	4	567208.601	141802.150	1.894	.1107
Column 14	2	8965646.620	4482823.310	59.880	<.0001
Column 11 * Column 14	8	1600119.702	200014.963	2.672	.0073
Residual	383	28672530.733	74863.005		

6724 cases were omitted due to missing values.

Means Table for Column 17
Effect: Column 11

	Count	Mean	Std. Dev.	Std. Err.
BI	79	561.823	315.179	35.460
INT	80	581.150	347.949	38.902
INT+ENT	80	539.475	265.909	29.730
SHAM	79	566.886	328.919	37.006
UNI	80	618.650	316.285	35.362

6724 cases were omitted due to missing values.

Interaction Bar Plot for Column 17
Effect: Column 11
Error Bars: 95% Confidence Interval

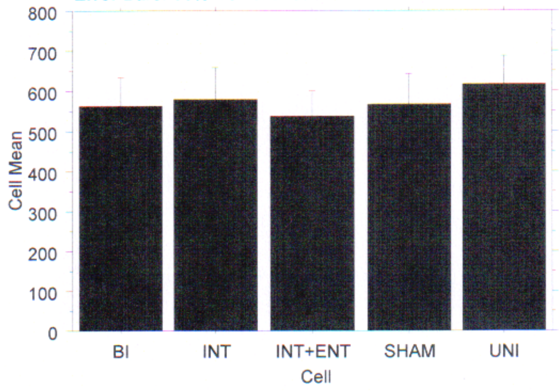

6724 cases were omitted due to missing values.

Bonferroni/Dunn for Column 17
Effect: Column 11
Significance Level: 5 %

	Mean Diff.	Crit. Diff	P-Value
BI, INT	-19.327	122.531	.6563
BI, INT+ENT	22.348	122.531	.6069
BI, SHAM	-5.063	122.915	.9075
BI, UNI	-56.827	122.531	.1912
INT, INT+ENT	41.675	122.145	.3360
INT, SHAM	14.264	122.531	.7426
INT, UNI	-37.500	122.145	.3866
INT+ENT, SH...	-27.411	122.531	.5280
INT+ENT, UNI	-79.175	122.145	.0680
SHAM, UNI	-51.764	122.531	.2337

Comparisons in this table are not significant unless the corresponding p-value is less than .005.
6724 cases were omitted due to missing values.

Means Table for Column 17
Effect: Column 11 * Column 14

	Count	Mean	Std. Dev.	Std. Err.
BI, S1	24	351.667	29.433	6.008
BI, S2	39	703.487	124.480	19.933
BI, S3	16	531.750	593.434	148.358
INT, S1	24	398.417	224.475	45.821
INT, S2	40	797.250	206.114	32.589
INT, S3	16	315.000	443.163	110.791
INT+ENT, S1	24	386.167	142.604	29.109
INT+ENT, S2	40	685.750	114.011	18.027
INT+ENT, S3	16	403.750	439.435	109.859
SHAM, S1	24	459.250	315.587	64.419
SHAM, S2	39	722.103	197.813	31.675
SHAM, S3	16	350.000	423.535	105.884
UNI, S1	24	430.750	165.019	33.684
UNI, S2	40	708.550	180.072	28.472
UNI, S3	16	675.750	565.045	141.261

6724 cases were omitted due to missing values.

Core Body Temperature
Duration
Anticipatory Component

ANOVA Table for Column 7

	DF	Sum of Squares	Mean Square	F-Value	P-Value
Column 2	4	193286.954	48321.739	7.262	<.0001
Column 5	2	37429.882	18714.941	2.813	.0613
Column 2 * Column 5	8	70468.137	8808.517	1.324	.2299
Residual	381	2535052.055	6653.680		

372 cases were omitted due to missing values.

Means Table for Column 7
Effect: Column 2

	Count	Mean	Std. Dev.	Std. Err.
BI	79	93.570	79.899	8.989
INT	80	154.150	86.473	9.668
INT+ENT	80	141.050	84.176	9.411
SHAM	79	157.975	85.076	9.572
UNI	78	150.667	74.731	8.462

372 cases were omitted due to missing values.

Interaction Bar Plot for Column 7
Effect: Column 2
Error Bars: 95% Confidence Interval

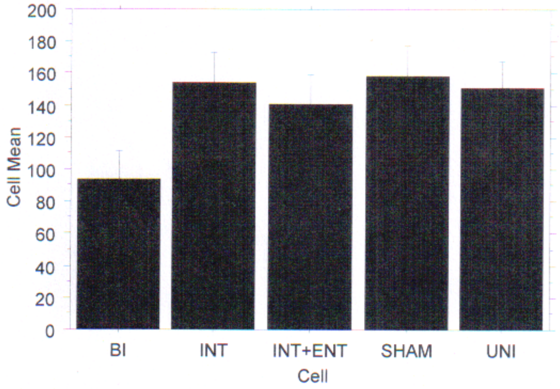

372 cases were omitted due to missing values.

Bonferroni/Dunn for Column 7
Effect: Column 2
Significance Level: 5 %

	Mean Diff.	Crit. Diff	P-Value	
BI, INT	-60.580	36.530	<.0001	S
BI, INT+ENT	-47.480	36.530	.0003	S
BI, SHAM	-64.405	36.645	<.0001	S
BI, UNI	-57.097	36.762	<.0001	S
INT, INT+ENT	13.100	36.415	.3104	
INT, SHAM	-3.825	36.530	.7677	
INT, UNI	3.483	36.648	.7886	
INT+ENT, SH...	-16.925	36.530	.1916	
INT+ENT, UNI	-9.617	36.648	.4592	
SHAM, UNI	7.308	36.762	.5749	

Comparisons in this table are not significant unless the corresponding p-value is less than .005.
372 cases were omitted due to missing values.

Means Table for Column 7
Effect: Column 2 * Column 5

	Count	Mean	Std. Dev.	Std. Err.
BI, S1	24	75.167	61.491	12.552
BI, S2	39	102.359	90.486	14.489
BI, S3	16	99.750	76.632	19.158
INT, S1	24	182.833	76.196	15.554
INT, S2	40	142.600	98.936	15.643
INT, S3	16	140.000	56.071	14.018
INT+ENT, S1	24	144.833	81.820	16.702
INT+ENT, S2	40	148.300	89.572	14.163
INT+ENT, S3	16	117.250	73.655	18.414
SHAM, S1	24	192.333	72.658	14.831
SHAM, S2	39	151.949	94.466	15.127
SHAM, S3	16	121.125	59.194	14.799
UNI, S1	23	159.304	79.285	16.532
UNI, S2	39	151.385	78.766	12.613
UNI, S3	16	136.500	58.330	14.583

372 cases were omitted due to missing values.

Core Body Temperature Intensity Anticipatory Component

ANOVA Table for Column 8

	DF	Sum of Squares	Mean Square	F-Value	P-Value
Column 2	4	1.268	.317	10.010	<.0001
Column 5	2	.210	.105	3.317	.0373
Column 2 * Column 5	8	.478	.060	1.887	.0607
Residual	382	12.097	.032		

371 cases were omitted due to missing values.

Means Table for Column 8
Effect: Column 2

	Count	Mean	Std. Dev.	Std. Err.
BI	79	.217	.159	.018
INT	80	.333	.194	.022
INT+ENT	80	.371	.183	.021
SHAM	79	.381	.191	.021
UNI	79	.325	.173	.019

371 cases were omitted due to missing values.

Interaction Bar Plot for Column 8
Effect: Column 2
Error Bars: 95% Confidence Interval

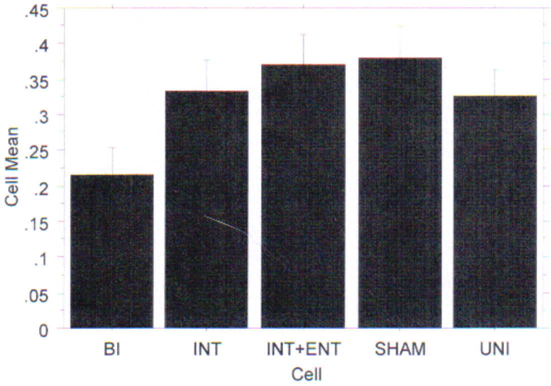

371 cases were omitted due to missing values.

Bonferroni/Dunn for Column 8
Effect: Column 2
Significance Level: 5 %

	Mean Diff.	Crit. Diff	P-Value	
BI, INT	-.117	.080	<.0001	S
BI, INT+ENT	-.154	.080	<.0001	S
BI, SHAM	-.164	.080	<.0001	S
BI, UNI	-.108	.080	.0002	S
INT, INT+ENT	-.037	.079	.1863	
INT, SHAM	-.048	.080	.0929	
INT, UNI	.009	.080	.7605	
INT+ENT, SH...	-.010	.080	.7156	
INT+ENT, UNI	.046	.080	.1050	
SHAM, UNI	.056	.080	.0481	

Comparisons in this table are not significant unless the corresponding p-value is less than .005.
371 cases were omitted due to missing values.

Means Table for Column 8
Effect: Column 2 * Column 5

	Count	Mean	Std. Dev.	Std. Err.
BI, S1	24	.175	.128	.026
BI, S2	39	.229	.173	.028
BI, S3	16	.249	.164	.041
INT, S1	24	.424	.188	.038
INT, S2	40	.301	.207	.033
INT, S3	16	.278	.113	.028
INT+ENT, S1	24	.384	.166	.034
INT+ENT, S2	40	.365	.200	.032
INT+ENT, S3	16	.363	.177	.044
SHAM, S1	24	.461	.192	.039
SHAM, S2	39	.329	.185	.030
SHAM, S3	16	.386	.170	.042
UNI, S1	24	.356	.223	.045
UNI, S2	40	.326	.162	.026
UNI, S3	15	.270	.090	.023

371 cases were omitted due to missing values.
