# Supplementary material for: Participation of the Olfactory Bulb in Circadian Organization during Early Postnatal Life in Rabbits
Source: PLoS One. 2016 Jun 15;11(6):e0156539. doi: 10.1371/journal.pone.0156539 (PMC4909232; doi:10.1371/journal.pone.0156539)
Supplement: S2 Text — Results of two-way ANOVA and Scheffe post-hoc test, of locomotor activity daily average, phases, and the duration and intensity of the anticipatory component, of intact rabbit pups, intact pups fed by enteral gavage, sham operated, with unilateral lesions of the olfactory bulb, and with bilateral lesions of the olfactory bulb. (PDF) [file pone.0156539.s004.pdf]

LOCOMOTOR ACTIVITY
Daily Average

ANOVA Table for Column 6

	DF	Sum of Squares	Mean Square	F-Value	P-Value
Column 2	4	121.613	30.403	3.942	.0038
Column 3	9	617.911	68.657	8.902	<.0001
Column 2 * Column 3	36	208.452	5.790	.751	.8517
Residual	344	2653.119	7.713		

365 cases were omitted due to missing values.

Means Table for Column 6
Effect: Column 2

	Count	Mean	Std. Dev.	Std. Err.
BI	79	12.288	3.237	.364
INT	80	13.063	2.617	.293
INT+ENT	76	12.503	2.437	.280
SHAM	79	12.309	2.938	.331
UNI	80	13.699	3.563	.398

365 cases were omitted due to missing values.

Interaction Bar Plot for Column 6
Effect: Column 2
Error Bars: 95% Confidence Interval

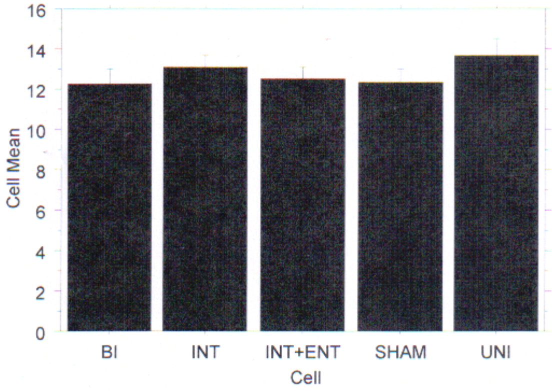

365 cases were omitted due to missing values.

Bonferroni/Dunn for Column 6
Effect: Column 2
Significance Level: 5 %

	Mean Diff.	Crit. Diff	P-Value	
BI, INT	-.775	1.245	.0794	
BI, INT+ENT	-.215	1.261	.6309	
BI, SHAM	-.020	1.248	.9633	
BI, UNI	-1.411	1.245	.0015	S
INT, INT+ENT	.560	1.257	.2086	
INT, SHAM	.755	1.245	.0875	
INT, UNI	-.636	1.241	.1487	
INT+ENT, SH...	.194	1.261	.6636	
INT+ENT, UNI	-1.196	1.257	.0075	
SHAM, UNI	-1.390	1.245	.0017	S

Comparisons in this table are not significant unless the corresponding p-value is less than .005.
365 cases were omitted due to missing values.

Means Table for Column 6
Effect: Column 2 * Column 3

	Count	Mean	Std. Dev.	Std. Err.
BI, P10	8	11.808	2.371	.838
BI, P11	8	10.979	2.650	.937
BI, P12	8	11.268	2.254	.797
BI, P13	7	10.838	2.136	.807
BI, P14	8	11.701	1.859	.657
BI, P15	8	13.345	2.647	.936
BI, P6	8	15.747	5.291	1.871
BI, P7	8	12.982	4.157	1.470
BI, P8	8	12.042	3.265	1.154
BI, P9	8	11.993	2.672	.945
INT, P10	8	12.672	2.896	1.024
INT, P11	8	12.863	2.494	.882
INT, P12	8	11.988	2.091	.739
INT, P13	8	12.055	2.404	.850
INT, P14	8	14.021	2.471	.873
INT, P15	8	11.398	3.077	1.088
INT, P6	8	14.841	3.148	1.113
INT, P7	8	13.473	2.209	.781
INT, P8	8	13.573	2.752	.973
INT, P9	8	13.749	1.738	.615
INT+ENT, P...	8	13.225	2.000	.707
INT+ENT, P...	8	11.624	1.714	.606
INT+ENT, P...	8	11.299	1.733	.613
INT+ENT, P...	8	10.935	1.566	.554
INT+ENT, P...	7	11.117	1.841	.696
INT+ENT, P...	6	10.154	1.142	.466
INT+ENT, P6	7	14.496	3.068	1.160
INT+ENT, P7	8	14.180	2.414	.854
INT+ENT, P8	8	13.251	1.173	.415
INT+ENT, P9	8	14.239	2.877	1.017
SHAM, P10	8	11.174	1.605	.567
SHAM, P11	8	9.928	1.307	.462
SHAM, P12	8	10.060	1.325	.469
SHAM, P13	7	10.575	1.570	.593
SHAM, P14	8	12.915	2.397	.848
SHAM, P15	8	12.251	3.300	1.167
SHAM, P6	8	15.895	3.387	1.197
SHAM, P7	8	14.671	3.692	1.305
SHAM, P8	8	12.973	2.019	.714
SHAM, P9	8	12.427	1.857	.657
UNI, P10	8	12.647	2.807	.993
UNI, P11	8	12.155	2.724	.963
UNI, P12	8	12.438	2.267	.802
UNI, P13	8	12.440	3.202	1.132
UNI, P14	8	15.093	3.144	1.112
UNI, P15	8	12.487	2.937	1.038
UNI, P6	8	16.498	4.832	1.708
UNI, P7	8	14.555	4.597	1.625
UNI, P8	8	14.123	3.766	1.332
UNI, P9	8	14.555	3.616	1.278

365 cases were omitted due to missing values.

LOCOMOTOR ACTIVITY
Achrophases

ANOVA Table for Column 13

	DF	Sum of Squares	Mean Square	F-Value	P-Value
Column 2	4	1306805.870	326701.468	3.933	.0039
Column 4	2	7877353.043	3938676.522	47.416	<.0001
Column 2 * Column 4	8	2603227.906	325403.488	3.917	.0002
Residual	348	28907196.678	83066.657		

396 cases were omitted due to missing values.

Means Table for Column 13
Effect: Column 2

	Count	Mean	Std. Dev.	Std. Err.
BI	79	669.443	427.761	48.127
INT	71	572.479	296.750	35.218
INT+ENT	72	576.194	360.385	42.472
SHAM	70	537.286	294.990	35.258
UNI	71	498.648	212.007	25.161

396 cases were omitted due to missing values.

Interaction Bar Plot for Column 13
Effect: Column 2
Error Bars: 95% Confidence Interval

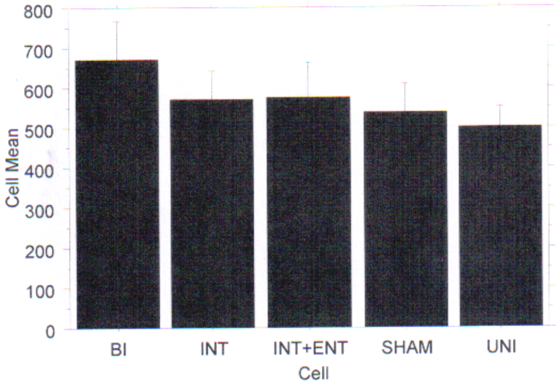

396 cases were omitted due to missing values.

Bonferroni/Dunn for Column 13
Effect: Column 2
Significance Level: 5 %

	Mean Diff.	Crit. Diff	P-Value	
BI, INT	96.964	133.150	.0404	
BI, INT+ENT	93.249	132.662	.0478	
BI, SHAM	132.157	133.650	.0055	
BI, UNI	170.795	133.150	.0003	S
INT, INT+ENT	-3.716	136.179	.9386	
INT, SHAM	35.193	137.142	.4690	
INT, UNI	73.831	136.655	.1278	
INT+ENT, SH...	38.909	136.668	.4218	
INT+ENT, UNI	77.547	136.179	.1086	
SHAM, UNI	38.638	137.142	.4266	

Comparisons in this table are not significant unless the corresponding p-value is less than .005.
396 cases were omitted due to missing values.

Means Table for Column 13
Effect: Column 2 * Column 4

	Count	Mean	Std. Dev.	Std. Err.
BI, S1	24	671.167	510.088	104.121
BI, S2	39	641.949	413.242	66.172
BI, S3	16	733.875	336.928	84.232
INT, S1	18	286.889	58.197	13.717
INT, S2	37	639.351	302.014	49.651
INT, S3	16	739.125	222.876	55.719
INT+ENT, S1	16	269.000	127.147	31.787
INT+ENT, S2	40	542.450	273.335	43.218
INT+ENT, S3	16	967.750	370.275	92.569
SHAM, S1	15	245.733	61.662	15.921
SHAM, S2	39	540.667	249.797	39.999
SHAM, S3	16	802.375	280.490	70.123
UNI, S1	19	298.526	149.187	34.226
UNI, S2	36	522.833	125.107	20.851
UNI, S3	16	681.875	242.036	60.509

396 cases were omitted due to missing values.

ANOVA Table for Column 14

	DF	Sum of Squares	Mean Square	F-Value	P-Value
Column 2	4	1072439.971	268109.993	2.008	.0927
Column 4	2	5919585.140	2959792.570	22.169	<.0001
Column 2 * Column 4	8	1237948.918	154743.615	1.159	.3230
Residual	383	51134891.751	133511.467		

361 cases were omitted due to missing values.

LOCOMOTOR ACTIVITY
Nadir

Means Table for Column 14
Effect: Column 2

	Count	Mean	Std. Dev.	Std. Err.
BI	79	633.873	331.083	37.250
INT	80	756.050	401.268	44.863
INT+ENT	80	710.975	443.124	49.543
SHAM	79	716.608	349.755	39.350
UNI	80	772.100	389.097	43.502

361 cases were omitted due to missing values.

Interaction Bar Plot for Column 14
Effect: Column 2
Error Bars: 95% Confidence Interval

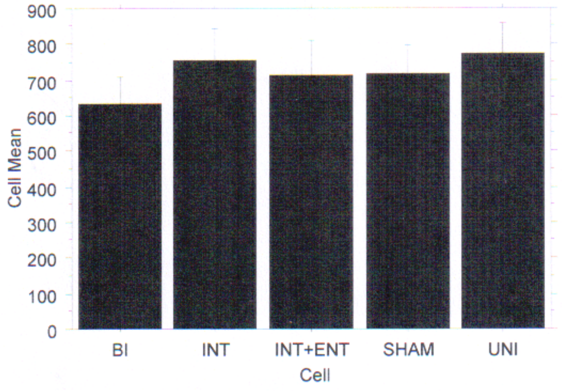

361 cases were omitted due to missing values.

Bonferroni/Dunn for Column 14
Effect: Column 2
Significance Level: 5 %

	Mean Diff.	Crit. Diff	P-Value
BI, INT	-122.177	163.633	.0357
BI, INT+ENT	-77.102	163.633	.1842
BI, SHAM	-82.734	164.147	.1555
BI, UNI	-138.227	163.633	.0176
INT, INT+ENT	45.075	163.117	.4358
INT, SHAM	39.442	163.633	.4966
INT, UNI	-16.050	163.117	.7813
INT+ENT, SH...	-5.633	163.633	.9226
INT+ENT, UNI	-61.125	163.117	.2907
SHAM, UNI	-55.492	163.633	.3389

Comparisons in this table are not significant unless the corresponding p-value is less than .005.
361 cases were omitted due to missing values.

Means Table for Column 14
Effect: Column 2 * Column 4

	Count	Mean	Std. Dev.	Std. Err.
BI, S1	24	549.833	292.433	59.693
BI, S2	39	777.026	244.795	39.199
BI, S3	16	411.000	411.709	102.927
INT, S1	24	632.833	354.841	72.432
INT, S2	40	843.350	374.765	59.256
INT, S3	16	722.625	493.943	123.486
INT+ENT, S1	24	728.167	431.057	87.989
INT+ENT, S2	40	809.400	418.676	66.199
INT+ENT, S3	16	439.125	434.003	108.501
SHAM, S1	24	557.417	235.522	48.076
SHAM, S2	39	851.487	276.428	44.264
SHAM, S3	16	626.625	510.872	127.718
UNI, S1	24	613.500	304.371	62.129
UNI, S2	40	916.250	332.355	52.550
UNI, S3	16	649.625	506.410	126.603

361 cases were omitted due to missing values.

LOCOMOTOR ACTIVITY
Duration
Anticipatory Component

ANOVA Table for Column 23

	DF	Sum of Squares	Mean Square	F-Value	P-Value
Column 18	4	32920.533	8230.133	2.776	.0269
Column 20	2	12632.876	6316.438	2.130	.1202
Column 18 * Column 20	8	37783.412	4722.926	1.593	.1251
Residual	383	1135682.621	2965.229		

361 cases were omitted due to missing values.

Means Table for Column 23
Effect: Column 18

	Count	Mean	Std. Dev.	Std. Err.
BI	79	57.190	44.216	4.975
INT	80	90.350	60.460	6.760
INT+ENT	80	87.100	59.474	6.649
SHAM	79	87.899	52.486	5.905
UNI	80	81.225	56.336	6.299

361 cases were omitted due to missing values.

Interaction Bar Plot for Column 23
Effect: Column 18
Error Bars: 95% Confidence Interval

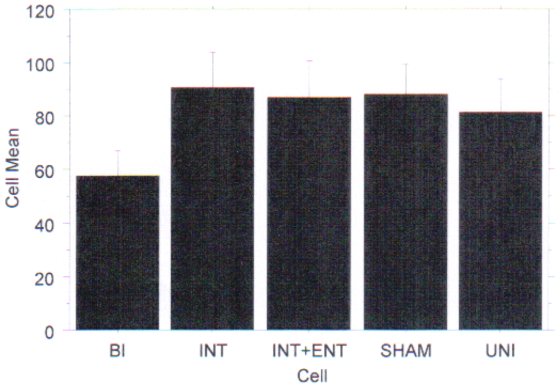

361 cases were omitted due to missing values.

Bonferroni/Dunn for Column 23
Effect: Column 18
Significance Level: 5 %

	Mean Diff.	Crit. Diff	P-Value	
BI, INT	-33.160	24.386	.0001	S
BI, INT+ENT	-29.910	24.386	.0006	S
BI, SHAM	-30.709	24.463	.0004	S
BI, UNI	-24.035	24.386	.0057	
INT, INT+ENT	3.250	24.309	.7060	
INT, SHAM	2.451	24.386	.7767	
INT, UNI	9.125	24.309	.2899	
INT+ENT, SH...	-.799	24.386	.9264	
INT+ENT, UNI	5.875	24.309	.4954	
SHAM, UNI	6.674	24.386	.4402	

Comparisons in this table are not significant unless the corresponding p-value is less than .005.
361 cases were omitted due to missing values.

Means Table for Column 23
Effect: Column 18 * Column 20

	Count	Mean	Std. Dev.	Std. Err.
BI, S1	24	58.000	42.570	8.689
BI, S2	39	51.385	39.818	6.376
BI, S3	16	70.125	55.711	13.928
INT, S1	24	76.750	47.996	9.797
INT, S2	40	109.600	66.787	10.560
INT, S3	16	62.625	44.928	11.232
INT+ENT, S1	24	72.083	51.498	10.512
INT+ENT, S2	40	97.650	64.364	10.177
INT+ENT, S3	16	83.250	55.837	13.959
SHAM, S1	24	95.750	45.524	9.293
SHAM, S2	39	87.231	58.302	9.336
SHAM, S3	16	77.750	48.232	12.058
UNI, S1	24	75.583	38.376	7.833
UNI, S2	40	85.900	66.151	10.459
UNI, S3	16	78.000	54.396	13.599

361 cases were omitted due to missing values.

LOCOMOTOR ACTIVITY
Duration
Anticipatory Component

Interaction Line Plot for Column 23
Effect: Column 21

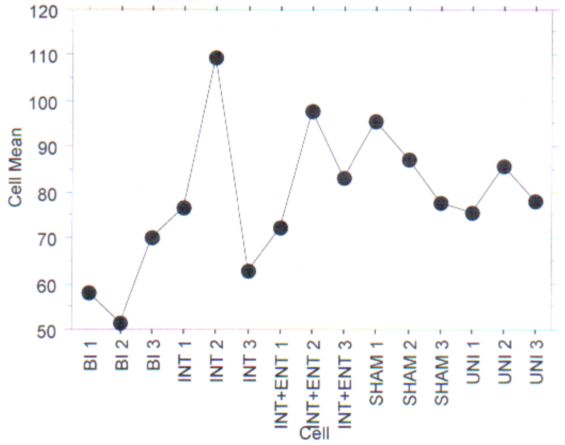

361 cases were omitted due to missing values.

Bonferroni/Dunn for Column 23
Effect: Column 21
Significance Level: 5 %

	Mean Diff.	Crit. Diff	P-Value	
BI 1, BI 2	6.615	49.787	.6399	
BI 1, BI 3	-12.125	61.937	.4907	
BI 1, INT 1	-18.750	55.398	.2337	
BI 1, INT 2	-51.600	49.549	.0003	S
BI 1, INT 3	-4.625	61.937	.7926	
BI 1, INT+ENT 1	-14.083	55.398	.3709	
BI 1, INT+ENT 2	-39.650	49.549	.0051	
BI 1, INT+ENT 3	-25.250	61.937	.1516	
BI 1, SHAM 1	-37.750	55.398	.0168	
BI 1, SHAM 2	-29.231	49.787	.0392	
BI 1, SHAM 3	-19.750	61.937	.2618	
BI 1, UNI 1	-17.583	55.398	.2640	
BI 1, UNI 2	-27.900	49.549	.0479	
BI 1, UNI 3	-20.000	61.937	.2558	
BI 2, BI 3	-18.740	56.974	.2471	
BI 2, INT 1	-25.365	49.787	.0734	
BI 2, INT 2	-58.215	43.185	<.0001	S
BI 2, INT 3	-11.240	56.974	.4873	
BI 2, INT+ENT 1	-20.699	49.787	.1437	
BI 2, INT+ENT 2	-46.265	43.185	.0002	S
BI 2, INT+ENT 3	-31.865	56.974	.0494	
BI 2, SHAM 1	-44.365	49.787	.0018	
BI 2, SHAM 2	-35.846	43.458	.0039	
BI 2, SHAM 3	-26.365	56.974	.1037	
BI 2, UNI 1	-24.199	49.787	.0875	
BI 2, UNI 2	-34.515	43.185	.0051	
BI 2, UNI 3	-26.615	56.974	.1005	
BI 3, INT 1	-6.625	61.937	.7064	
BI 3, INT 2	-39.475	56.766	.0147	
BI 3, INT 3	7.500	67.848	.6971	
BI 3, INT+ENT 1	-1.958	61.937	.9113	
BI 3, INT+ENT 2	-27.525	56.766	.0883	
BI 3, INT+ENT 3	-13.125	67.848	.4958	
BI 3, SHAM 1	-25.625	61.937	.1456	
BI 3, SHAM 2	-17.106	56.974	.2907	

BI 3, SHAM 3	-7.625	67.848	.6923
BI 3, UNI 1	-5.458	61.937	.7563
BI 3, UNI 2	-15.775	56.766	.3280
BI 3, UNI 3	-7.875	67.848	.6827
INT 1, INT 2	-32.850	49.549	.0200
INT 1, INT 3	14.125	61.937	.4221
INT 1, INT+ENT 1	4.667	55.398	.7667
INT 1, INT+ENT 2	-20.900	49.549	.1380
INT 1, INT+ENT 3	-6.500	61.937	.7117
INT 1, SHAM 1	-19.000	55.398	.2275
INT 1, SHAM 2	-10.481	49.787	.4586
INT 1, SHAM 3	-1.000	61.937	.9547
INT 1, UNI 1	1.167	55.398	.9409
INT 1, UNI 2	-9.150	49.549	.5156
INT 1, UNI 3	-1.250	61.937	.9433
INT 2, INT 3	46.975	56.766	.0038
INT 2, INT+ENT 1	37.517	49.549	.0079
INT 2, INT+ENT 2	11.950	42.911	.3270
INT 2, INT+ENT 3	26.350	56.766	.1027
INT 2, SHAM 1	13.850	49.549	.3252
INT 2, SHAM 2	22.369	43.185	.0687
INT 2, SHAM 3	31.850	56.766	.0487
INT 2, UNI 1	34.017	49.549	.0160
INT 2, UNI 2	23.700	42.911	.0523
INT 2, UNI 3	31.600	56.766	.0505
INT 3, INT+ENT 1	-9.458	61.937	.5908
INT 3, INT+ENT 2	-35.025	56.766	.0303
INT 3, INT+ENT 3	-20.625	67.848	.2847
INT 3, SHAM 1	-33.125	61.937	.0602
INT 3, SHAM 2	-24.606	56.974	.1288
INT 3, SHAM 3	-15.125	67.848	.4326
INT 3, UNI 1	-12.958	61.937	.4614
INT 3, UNI 2	-23.275	56.766	.1493
INT 3, UNI 3	-15.375	67.848	.4250
INT+ENT 1, INT+EN...	-25.567	49.549	.0698
INT+ENT 1, INT+EN...	-11.167	61.937	.5256
INT+ENT 1, SHAM 1	-23.667	55.398	.1330
INT+ENT 1, SHAM 2	-15.147	49.787	.2843
INT+ENT 1, SHAM 3	-5.667	61.937	.7473
INT+ENT 1, UNI 1	-3.500	55.398	.8239
INT+ENT 1, UNI 2	-13.817	49.549	.3264
INT+ENT 1, UNI 3	-5.917	61.937	.7366
INT+ENT 2, INT+EN...	14.400	56.766	.3719
INT+ENT 2, SHAM 1	1.900	49.549	.8926
INT+ENT 2, SHAM 2	10.419	43.185	.3957
INT+ENT 2, SHAM 3	19.900	56.766	.2174
INT+ENT 2, UNI 1	22.067	49.549	.1174
INT+ENT 2, UNI 2	11.750	42.911	.3352
INT+ENT 2, UNI 3	19.650	56.766	.2232
INT+ENT 3, SHAM 1	-12.500	61.937	.4774
INT+ENT 3, SHAM 2	-3.981	56.974	.8056
INT+ENT 3, SHAM 3	5.500	67.848	.7753
INT+ENT 3, UNI 1	7.667	61.937	.6629
INT+ENT 3, UNI 2	-2.650	56.766	.8694
INT+ENT 3, UNI 3	5.250	67.848	.7852
SHAM 1, SHAM 2	8.519	49.787	.5468
SHAM 1, SHAM 3	18.000	61.937	.3064
SHAM 1, UNI 1	20.167	55.398	.2003
SHAM 1, UNI 2	9.850	49.549	.4840
SHAM 1, UNI 3	17.750	61.937	.3132

SHAM 2, SHAM 3	9.481	56.974	.5579
SHAM 2, UNI 1	11.647	49.787	.4102
SHAM 2, UNI 2	1.331	43.185	.9136
SHAM 2, UNI 3	9.231	56.974	.5683
SHAM 3, UNI 1	2.167	61.937	.9019
SHAM 3, UNI 2	-8.150	56.766	.6132
SHAM 3, UNI 3	-.250	67.848	.9896
UNI 1, UNI 2	-10.317	49.549	.4635
UNI 1, UNI 3	-2.417	61.937	.8907
UNI 2, UNI 3	7.900	56.766	.6241

Comparisons in this table are not significant unless the corresponding p-value is less than .0005.
361 cases were omitted due to missing values.

LOCOMOTOR ACTIVITY
Intensity
Anticipatory Component

ANOVA Table for Column 8

	DF	Sum of Squares	Mean Square	F-Value	P-Value
Column 2	4	649.969	162.492	3.737	.0054
Column 4	2	384.460	192.230	4.421	.0126
Column 2 * Column 4	8	160.320	20.040	.461	.8833
Residual	380	16522.085	43.479		

364 cases were omitted due to missing values.

Means Table for Column 8
Effect: Column 2

	Count	Mean	Std. Dev.	Std. Err.
BI	79	11.778	6.478	.729
INT	80	14.330	7.012	.784
INT+ENT	80	12.663	5.608	.627
SHAM	79	13.218	5.100	.574
UNI	77	15.437	8.423	.960

364 cases were omitted due to missing values.

Interaction Bar Plot for Column 8
Effect: Column 2
Error Bars: 95% Confidence Interval

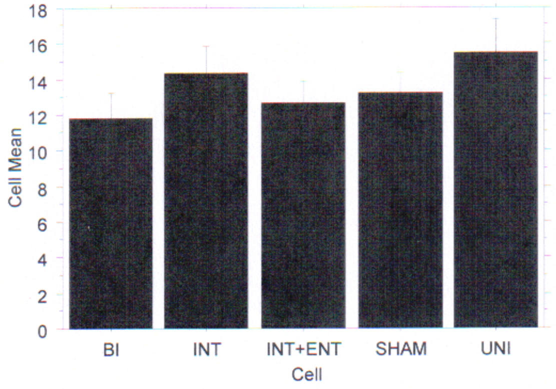

364 cases were omitted due to missing values.

Fisher's PLSD for Column 8
Effect: Column 2
Significance Level: 5 %

	Mean Diff.	Crit. Diff	P-Value	
BI, INT	-2.551	2.056	.0152	S
BI, INT+ENT	-.885	2.056	.3980	
BI, SHAM	-1.440	2.063	.1707	
BI, UNI	-3.659	2.076	.0006	S
INT, INT+ENT	1.666	2.050	.1108	
INT, SHAM	1.112	2.056	.2885	
INT, UNI	-1.108	2.070	.2934	
INT+ENT, SH...	-.555	2.056	.5961	
INT+ENT, UNI	-2.774	2.070	.0088	S
SHAM, UNI	-2.219	2.076	.0363	S

364 cases were omitted due to missing values.

Means Table for Column 8
Effect: Column 4

	Count	Mean	Std. Dev.	Std. Err.
S1	120	12.012	6.070	.554
S2	198	14.049	6.952	.494
S3	77	14.281	6.712	.765

364 cases were omitted due to missing values.

Means Table for Column 8
Effect: Column 2 * Column 4

	Count	Mean	Std. Dev.	Std. Err.
BI, S1	24	10.649	5.934	1.211
BI, S2	39	12.523	7.353	1.177
BI, S3	16	11.658	4.869	1.217
INT, S1	24	11.378	6.662	1.360
INT, S2	40	15.452	6.732	1.064
INT, S3	16	15.953	7.279	1.820
INT+ENT, S1	24	11.050	4.848	.990
INT+ENT, S2	40	13.306	5.618	.888
INT+ENT, S3	16	13.477	6.448	1.612
SHAM, S1	24	12.861	4.998	1.020
SHAM, S2	39	13.315	4.903	.785
SHAM, S3	16	13.517	5.977	1.494
UNI, S1	24	14.123	7.336	1.497
UNI, S2	40	15.594	9.126	1.443
UNI, S3	13	17.380	8.241	2.286

364 cases were omitted due to missing values.
